# Order of statistical learning depends on perceptive uncertainty

**DOI:** 10.1016/j.crneur.2023.100080

**Published:** 2023-03-01

**Authors:** Tatsuya Daikoku, Masato Yumoto

**Affiliations:** a International Research Center for Neurointelligence, The University of Tokyo, 7-3-1 Hongo, Bunkyo-ku, Tokyo, Japan; b Center for Brain, Mind and KANSEI Sciences Research, Hiroshima University, Hiroshima, Japan; c Advanced Medical Science Research Center, Gunma Paz University, Gunma, Japan

**Keywords:** entropy, Predictive coding, Information theory, Markovian, N-gram, ANOVA, analysis of variance, ERF, event-related magnetic fields, ERP, event-related potentials, ECDs, equivalent current dipoles, MEG, magnetoencephalography, MMN, mismatch-negativity, SL, statistical learning, TP, transition probability

## Abstract

Statistical learning (SL) is an innate mechanism by which the brain automatically encodes the *n*-th order transition probability (TP) of a sequence and grasps the uncertainty of the TP distribution. Through SL, the brain predicts a subsequent event (*e*_*n+1*_) based on the preceding events (*e*_*n*_) that have a length of “*n”*. It is now known that uncertainty modulates prediction in top-down processing by the human predictive brain. However, the manner in which the human brain modulates the order of SL strategies based on the degree of uncertainty remains an open question. The present study examined how uncertainty modulates the neural effects of SL and whether differences in uncertainty alter the order of SL strategies. It used auditory sequences in which the uncertainty of sequential information is manipulated based on the conditional entropy. Three sequences with different TP ratios of 90:10, 80:20, and 67:33 were prepared as low-, intermediate, and high-uncertainty sequences, respectively (conditional entropy: 0.47, 0.72, and 0.92 bit, respectively). Neural responses were recorded when the participants listened to the three sequences. The results showed that stimuli with lower TPs elicited a stronger neural response than those with higher TPs, as demonstrated by a number of previous studies. Furthermore, we found that participants adopted higher-order SL strategies in the high uncertainty sequence. These results may indicate that the human brain has an ability to flexibly alter the order based on the uncertainty. This uncertainty may be an important factor that determines the order of SL strategies. Particularly, considering that a higher-order SL strategy mathematically allows the reduction of uncertainty in information, we assumed that the brain may take higher-order SL strategies when encountering high uncertain information in order to reduce the uncertainty. The present study may shed new light on understanding individual differences in SL performance across different uncertain situations.

## Highlights

• Neural basis of statistical learning and its modulation by uncertainty was examined.
• Stimuli with lower transition probabilities elicit stronger neural responses.
• Uncertainty fine-tunes the computational algorithm of statistical learning in the brain.

## Graphical abstract

**Figure:**
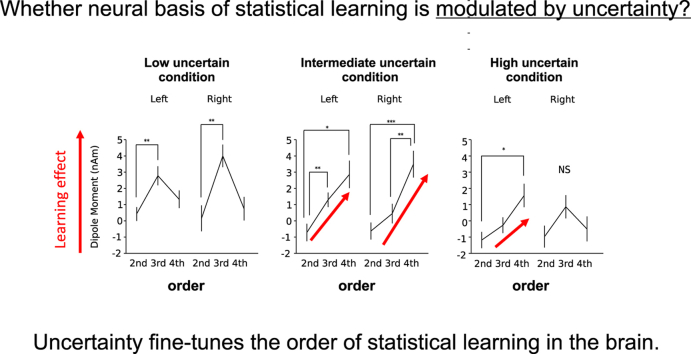

## 1 Introduction

### 1.1 Statistical learning and uncertainty

The brain models external environments as dynamic systems that encode probability distributions over states in the world. Based on the internalised probabilistic model, it expects an upcoming state and optimises actions by updating the model to resolve “*uncertainty*” in the external environment (Friston, 2010). Within such a predictive-processing framework, the precision and optimisation of predictability mandates the suppression of prediction errors and uncertainty.

This predictive processing underlies an innate function of the brain called statistical learning (SL) (Saffran et al., 1996). By implementing SL, the brain automatically encodes the *n*-th order transition probability (TP) of sequential information (Cleeremans et al., 1998; Perruchet and Pacton, 2006; Saffran et al., 1996; Daikoku, 2019) and grasps the uncertainty of TP distribution (Hasson, 2017). By SL of the sequential information, the brain predicts a subsequent event (*e*_*n+1*_) based on the preceding events that have the length of “*n”* (*e*_*n*_), written by a formula of *P(e*_*n+1*_*|e*_*n*_*)*. The brain's SL is thought to contribute to the acquisition of various types of sequential knowledge with complex structure such as language, music, action, and social communication (Monroy et al., 2017; Pearce and Wiggins, 2012; Peretz et al., 2012; Saffran et al., 1996). Thus, SL is an inherent and indispensable apparatus in the developing brain to learn and adapt to various types of uncertain information.

To precisely predict individual events in sequences, the brain also encodes the uncertainty of the TP distributions as well as each TP value. The uncertainty can be evaluated using “*entropy*” based on information theory (Daikoku et al., 2018) as done by Shannon (1948). In particular, within the framework of SL, conditional entropy (*H(X*_*n+1*_*|X*_*n*_*)*) can be used and is calculated from the *n*-th order TP distribution (Daikoku, 2018d). From a psychological viewpoint, a sequence with higher conditional entropy (i.e., a high uncertainty sequence) is regarded as sequential information that makes it more difficult for the human brain to implement SL and precisely predict individual events in sequences. Evidence from previous studies has also revealed that the degree of uncertainty fine-tunes the predictability of individual events in sequences during SL (Agres et al., 2018; Nastase et al., 2014). Further, the neural mechanisms underlying SL can also be modulated by the degree of uncertainty (Harrison et al., 2006; Hasson, 2017; Okano et al., 2021).

### 1.2 Order of statistical learning

Both neural and modelling studies have suggested the importance of the “order” of SL strategies (Du and Kelly, 2013; Daikoku, 2018c; 2019). The concept of order corresponds to the length of the preceding events (*e*_*n*_) to predict a subsequent event (*e*_*n+1*_) as expressed in the formula of TP, *P(e*_*n+1*_*|e*_*n*_*).* The value of order (i.e., "*n*") represents the SL strategies of how many past events are referenced to predict the next. For example, if the current stimulus is presented in uppercase, then responses to the expected transition in the streak 'expected- > expected- > Expected’ should be different than when it is located in the streak 'unexpected- > expected- > Expected’. More simply, if an individual learned these sequences by SL based on first-order TP (hereafter, first-order SL), they would not be able to distinguish between the two because the preceding one event and predicted event are the same as each other (expected- > Expected). However, if a person learned by second-order SL, the first event in the preceding two events is "'expected" for the former (‘expected- > expected- > Expected’) and "unexpected" for the latter (‘unexpected- > expected- > Expected’), so the person can distinguish between them. This would be evidence that responses to the current stimulus (Capitalised) is determined by a relatively long temporal integration window or higher-order SL.

### 1.3 Interdependence between uncertainty of information and order of statistical learning strategies

The order of SL strategies is not independent, but rather interdependent on the degree of uncertainty. In the information theory framework, higher-order SL models represent lower conditional entropy (i.e., uncertainty) (see Fig. 3b in Daikoku, 2018c). Even if the target information of SL is the same, the uncertainty changes depending on the order of SL strategies. However, it remains unknown whether the actual human brain can also modulate the order of SL strategies based on the degree of uncertainty in sequential information.

Most neural and psychological studies have focused on the specific order of SL strategies. For example, the word-segmentation paradigm is typically used to examine SL mechanisms (for review, see Daikoku, 2018c). This paradigm consists of a concatenation of pseudo-words, and the TPs of a word-segmentation task can be calculated based on first-order models. The SL of word segmentation based on first-order TPs is considered an inherent apparatus for language acquisition in the early stages of language learning, even in an immature infant brain (Saffran et al., 1996). Mounting evidence, however, indicates that SL machinery persists in the mature adult brain and that there may be various developmental stages of SL strategies. In fact, the first-order SL of word-segmentation that has typically been discussed in most studies is too simple and therefore could not explain learning mechanisms of more complex sequential structure like languages and music. The modulation of the “order” of SL strategies may be one of the important mechanisms to understand the hallmark of human SL function.

Importantly, the suppression and precision of uncertainty are a key role in our predictive brain (Friston, 2010). SL mechanisms contributes to this role: long-term SL may allow the brain to precisely predict a future event and to suppress and optimise uncertainty (Hansen and Pearce, 2014). Additionally, given the information-theoretical concept that the uncertainty (conditional entropy) is decreased in a higher-order SL strategy compared with a lower order of SL strategy, we hypothesised that the human brain also has a potential function to alter the “order” of SL strategies based on the uncertainty: the brain may adopt higher-order SL strategies in high uncertainty information in order to reduce the uncertainty. Such a flexible alteration of “order” may be essential for explaining more complex SL functions.

### 1.4 Neural probes of statistical learning

A number of experiments indicate that SL is reflected in neural as well as behavioural responses (reviewed in Daikoku, 2018c). For example, electroencephalography and magnetoencephalography (MEG) can directly measure neural activity during SL and represent a more sensitive method than the observation of behavioural effects (Koelsch et al., 2016; Paraskevopoulos et al., 2012). In the framework of predictive coding (Friston, 2005), when the brain encodes the *n*-th order TP distribution of stimulus sequences, it expects an upcoming stimulus (n+1) with a high TP based on the latest states (n). The brain suppresses the neural activity if the actual stimulus is the predicted one, whereas they are likely to be surprised if it is an unpredicted one (i.e., a stimulus with lower TP). Finally, the SL effect is revealed as a difference in neural activity between predictable and unpredictable stimuli.

A body of evidence has shown that such a SL effect is reflected in the event-related potentials (ERP) and event-related magnetic fields (ERF). In particular, the suppression of the components of the N1 (i.e., N100, around 100 ms after stimulus onset) and mismatch-negativity (MMN, around 100–200 ms after stimulus onset) of auditory responses to stimuli with a higher TP in lower cortical areas has been a typical neural marker to evaluate auditory SL effects (Daikoku, 2018c; Skoe et al., 2015; Koelsch et al., 2016). In the present study, we also targeted N1 components as a neural marker of SL and investigated how the neural effects of *n*-th order SL are distinctly represented in different uncertainty conditions.

### 1.5 The aim of the present study

The present study examined how the uncertainty of sequence modulates the neural effects of SL reflected in N1 components and whether the difference of uncertainty alters the order of SL strategies, using auditory sequences in which the uncertainty of sequential information is manipulated based on the conditional entropy. The three sequences with different TP ratios of 90:10, 80:20, and 67:33 were prepared as the low-, intermediate, and the high-uncertainty sequences, respectively (conditional entropy: 0.47, 0.72, and 0.92 bit, respectively). Neural responses were recorded when the participants listened to the three sequences. First, we hypothesised that stimuli with lower TPs would elicit a stronger neural response than those with higher TPs, as demonstrated by a number of previous studies based on predictive coding. To understand how uncertainty modulates the order of SL strategies, not only as 1st- but also as 2nd-, 3rd-, and 4th-order, TP distributions were re-calculated in each of the three sequences. We hypothesised that difference of uncertainty allows the brain to alter the order of the SL strategy. Particularly, considering that a higher-order SL strategy allows the reduction of uncertainty in information, we hypothesised that the brain may adopt higher-order SL strategies in high uncertainty information in order to reduce the uncertainty.

## 2 Methods

### 2.1 Participants

Sixteen right-handed (Edinburgh handedness questionnaires; laterality quotient ranged from 57.9 to 100) (Oldfield, 1971) healthy participants with no history of neurological or audiological disorders were included (9 men, 6 women; age range, 24–36 years). None of the participants had an absolute pitch. This study was approved by the Ethics Committee of the University of Tokyo and was performed in accordance with the guidelines and regulations. All participants were well informed of the purpose, safety, and protection of their personal data in this experiment, and they provided written informed consent for this study.

### 2.2 Stimuli

**Fig. 1:**
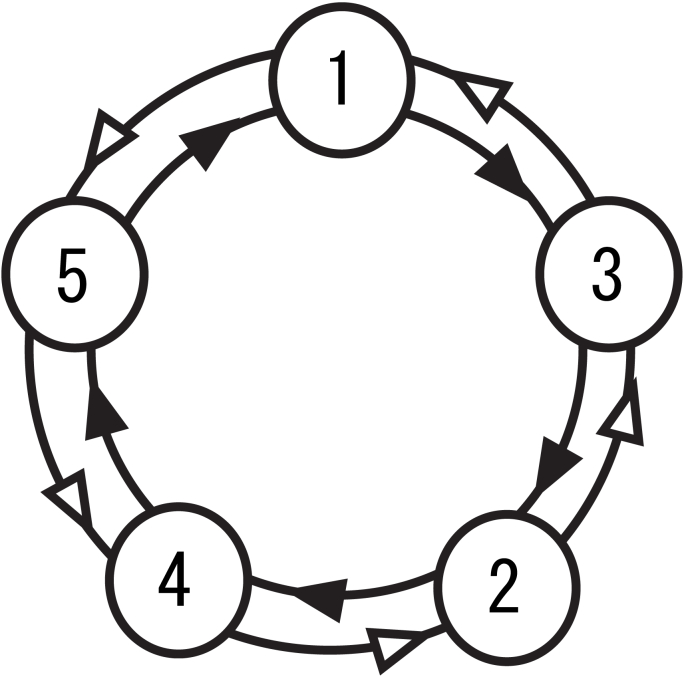
The transition diagram of the Markov chain used in the study. The tone could follow the most recent tone based on a higher or lower TP ratio. The two-tone sequences with different TP ratios of 90:10, 80:20, and 67:33 were prepared as the low-uncertain (conditional entropy = 0.47), intermediate (conditional entropy = 0.72), and the high-uncertain sequences (conditional entropy = 0.92), respectively.

Using five pure tones with equal amplitude and frequencies in a five-tone equal temperament (F0 = 350 × 2(n-1)/5 Hz, n = 1–5; 350, 402, 462, 531, and 609 Hz), we devised three sequences with 1500 tones, which were presented with a constant stimulus onset asynchrony (SOA) of 0.6 s (duration, 300 ms; rise/fall, 10/200 ms; binaural presentation, 80 dBSPL intensity). The order of pure tones was defined according to first-order Markov models (i.e., bi-gram model) with the constraint that the probability of a forthcoming tone was statistically defined by the most recent tone (Fig. 1). The two-tone sequences with different TP ratios of 90:10, 80:20, and 67:33, in which each conditional entropy in the probability distribution of sequences was 0.47, 0.72, and 0.92, respectively, were prepared as low-, intermediate-, and high-uncertainty sequences, respectively. Three distinct types of Markov chains were used in each of the three sequences and the use of Markov chains was counterbalanced across participants. The TPs of the sequences produced by these Markov processes exactly matched the target TPs and the conditional entropy (see OSF site: https://osf.io/xcr97/for the information of three sequences). A 600-ms silent period was pseudo-randomly inserted within every set of 50 successive tones in the sequence, and the participants were instructed to raise their right hand at every silent period in the sequence to confirm that they had attended to the novel tone sequences.

### 2.3 Measurement

Measurements and analyses were performed as described in our previous study (Daikoku et al., 2014, Daikoku et al., 2015, Daikoku et al., 2016). We recorded MEG signals from the participants while they listened to the three sequences. The order of the three sequences was counterbalanced across participants to ensure that specific transitional patterns did not interfere with learning in the adjacent regions. Auditory stimuli were sequenced using the STIM2 system (Compumedics Neuroscan, El Paso, TX, USA) and binaurally delivered to the participants’ ears at 80 dB SPL through ER-3A earphones (Etymotic Research, Elk Grove Village, IL, USA). MEG signals were recorded in a magnetically shielded room using a 306-channel neuromagnetometer system (Elekta Neuromag Oy, Helsinki, Finland) with 204 planar first-order gradiometers and 102 magnetometers at 102 measurement sites on a helmet-shaped surface covering the entire scalp. Auditory stimulus-triggered epochs were filtered online with a 0.1–200 Hz band-pass filter and recorded at a sampling rate of 600 Hz.

### 2.4 Data analysis

#### 2.4.1 Equivalent current dipoles (ECDs) estimation

Epochs with artifacts exceeding 3 pT/cm or 3 pT for any MEG channel were excluded from the analyses. Contamination from environmental noise was reduced using the temporally extended signal space separation method with a buffer length of 10 s and correlation limit of 0.980 (Taulu and Hari, 2009). All responses to the tones in the three sequences were averaged for each participant to evaluate the reliability of the evoked response components. The averaged responses were filtered offline with a 2–40 Hz band-pass filter. The baseline for magnetic signals in each MEG channel was defined by the mean amplitude in the pre-stimulus period from −100 to 0 ms. The analysis window was defined as 0–500 ms. The ECDs for the N1m responses in each hemisphere were separately estimated at peak latency using 66 temporal channels for each participant. Participants who demonstrated poor ECD estimation, with a goodness-of-fit below 80% in either the left or right hemisphere, were not used in further analysis. Ultimately, 13 participants demonstrated ECD estimation with a goodness-of-fit above 80%.

#### 2.4.2 Source-strength waveform and statistical analysis

##### 2.4.2.1 Analysis 1: comparison between higher and lower TP ratios

Selective response averaging was conducted separately for the first, middle, and last phases of the sequence, and was distinguished according to higher and lower TP based on the TP ratio, respectively (Fig. 1). The source-strength waveforms for N1m in each hemisphere were calculated using the ECDs as templates. We performed a 2 (*“Hemisphere”*: right and left) × 3 (*“Phase”*: first, middle, and last) × 2 (*“Probability”*: high and low) × 3 (*“Sequence”*: low-, intermediate-, and high-uncertainty sequences) repeated-measures analysis of variance (ANOVA) with the peak amplitude and latency of the source strength of N1m. Furthermore, we performed a 2 (*“Hemisphere”*: right and left) × 3 (*“Phase”*: first, middle, and last) × 3 (*“Sequence”*: low-, intermediate-, and high-uncertainty sequences) repeated-measures ANOVA with differences in amplitudes that were subtracted from the peak amplitudes for tones with high TP from those with low TP. When significant effects were detected, a Bonferroni-corrected post-hoc test was conducted for further analysis.

##### 2.4.2.2 Analysis 2: comparison among 2nd-, 3rd, and 4th-order transitions

**Fig. 2:**
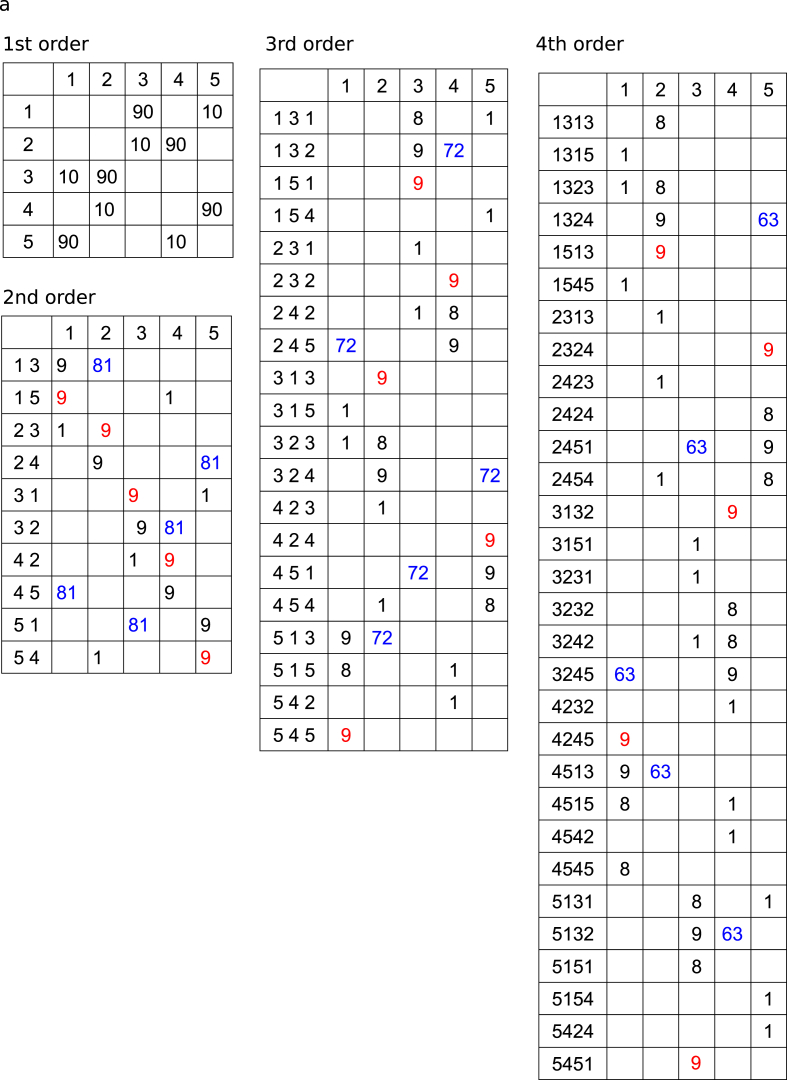

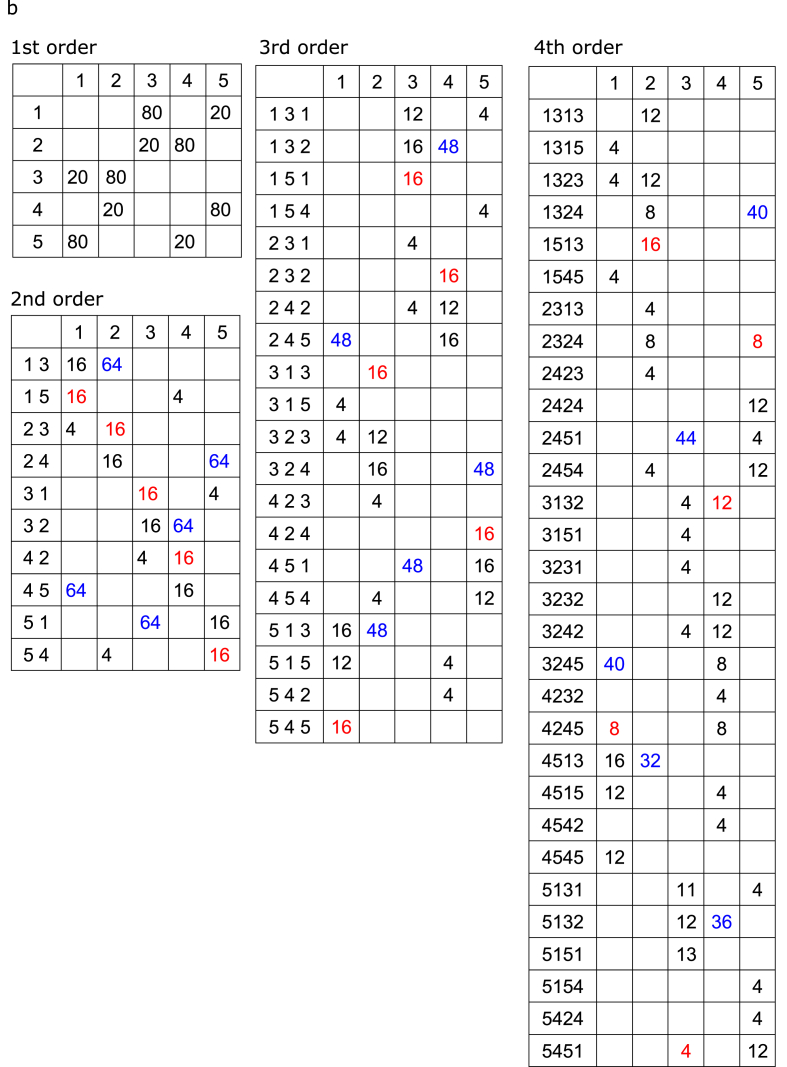

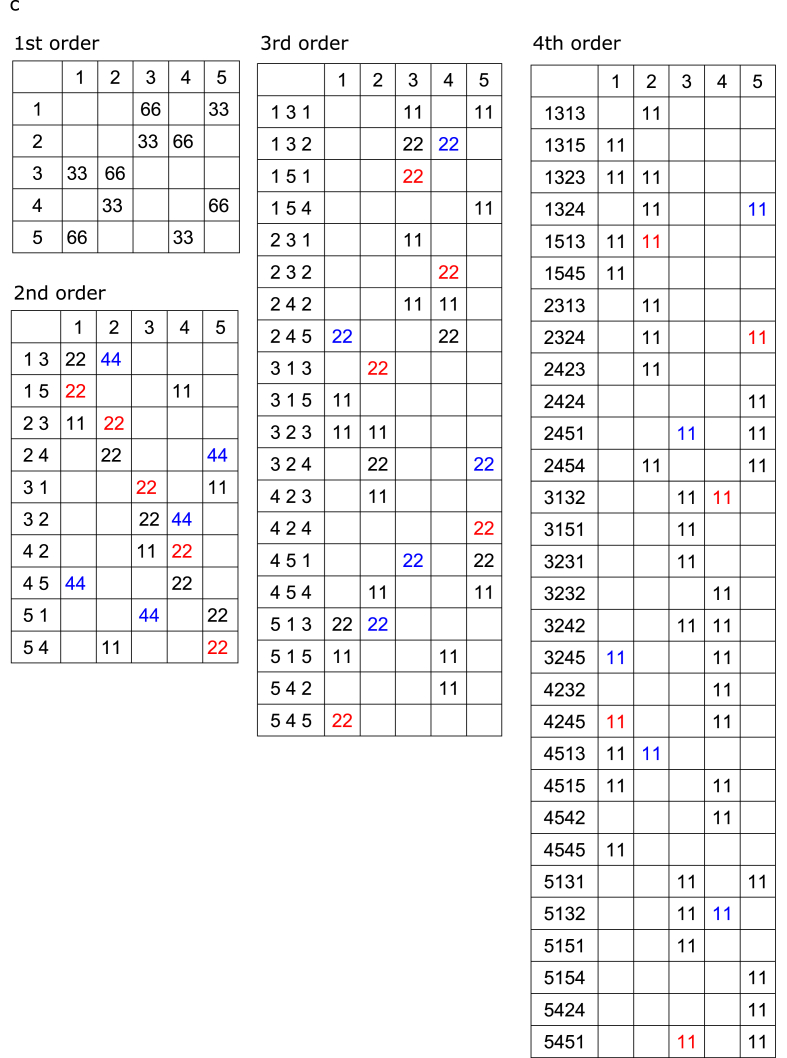
The matrices of the number of occurrence of transitions in a phase of the low- (a), intermediate- (b), and the high-uncertainty (c) sequences. The tone sequences were regarded not only as 1st- but also as 2nd-, 3rd-, and 4th-order Markov chains. The neural response to the tones with high (blue) and low (red) probabilities of occurrences in two transition patterns that were solely distinct first tones, were selectively averaged and compared, respectively. (For interpretation of the references to colour in this figure legend, the reader is referred to the Web version of this article.)

The three tone sequences were regarded as 1st- but also as 2nd-, 3rd-, and 4th-order Markov models (i.e., tri-gram, 4-g, and 5-g models, respectively). The matrices of the number of occurrences and conditional entropy in each Markov model are shown in Fig. 2 and Table 1, respectively. Selective response averaging was conducted separately for the first, middle, and last phases of the sequence and was distinguished according to the tones that were transitioned based on the two types of transition patterns with solely distinct first tones. The source-strength waveforms for N1m in each hemisphere were calculated using the ECDs as templates. Similar to Analysis 1, we performed a 2 (*“Hemisphere”*: right and left) × 3 (*“Phase”*: first, middle, and last) × 2 (*“Probability”*: high and low) × 3 (sequence: low-, intermediate-, and high-uncertainty sequences) repeated-measures ANOVA with peak amplitude and latency of source strength of N1m. Furthermore, we performed a 2 (*“Hemisphere”*: right and left) × 3 (*“Phase”*: first, middle, and last) × 3 (*“Sequence”*: low-, intermediate-, and high-uncertainty sequences) × 3 (*“Order”*: 2nd-, 3rd-, 4th-order Markov chains) repeated-measures ANOVA with differences in amplitudes that were subtracted from the peak amplitudes of a type of transition (red in Fig. 2) from those of the other types of transitions (blue in Fig. 2). When significant effects were detected, a Bonferroni-corrected post-hoc test was conducted for further analysis. Statistical significance was set at *p* = .05.

**Table 1:** Conditional entropy of TP distribution based on nth-order Markov model in low-, intermediate-, and high-uncertainty sequences.

	Low	Intermediate	High
1^st^	0.47	0.72	0.92
2^nd^	0.47 (1)	0.72 (1)	0.92 (1)
3^rd^	0.45 (0.97)	0.65 (0.90)	0.67 (0.73)
4^th^	0.44 (0.93)	0.58 (0.80)	0.677 (0.73)

## 3 Results

### 3.1 Uncertainty and SL

We performed a 2 (hemisphere: right and left) × 3 (phase: first, middle, and last) × 2 (probability: high and low in Fig. 1) × 3 (sequence: low-uncertain, intermediate, and high-uncertain sequences) repeated-measures ANOVA with the peak amplitude and latency of the source strength of N1m. The main phase effect on peak amplitudes and latencies was significant (amplitudes *F*[2, 24] = 19.37, *p* = .0001; latencies *F*[2, 24] = 35.81, *p* = .0001). The peak amplitudes were significantly lower in the last phase than in the first and middle phases (first: *p* = .001; middle: *p* = .039) and less in the middle phase than in the first phase (*p* = .001). Peak latencies were significantly longer in the first phase than in the middle and last phases (middle: *p* = .0001; last: *p* = .0001). The main probability effect on peak amplitudes and latencies was significant (*F*[1, 12] = 12.84, *p* = .004; latencies *F*[1, 12] = 6.86, *p* = .022). The peak amplitudes and latencies for tones with low TP were significantly larger and longer, respectively, than those with high TP. The main hemisphere effect on peak amplitudes was significant (*F*[1, 12] = 22.85, *p* = .0001). Peak amplitudes were significantly larger in the right hemisphere than in the left hemisphere. The hemisphere-phase interactions of the peak amplitudes were significant (*F*[2, 24] = 4.03, *p* = .031). The peak amplitudes were significantly lower in the middle and last phases than in the first phase in the left (middle: *p* = .006; last: *p* = .010) and right hemispheres (middle: *p* = .003; last: *p* = .002). Peak amplitudes were significantly lower in the last phase than in the middle phase in the right hemisphere (*p* = .017). The peak amplitudes were significantly larger in the right hemisphere than in the left hemisphere in the first (*p* = .0001), middle (*p* = .0001), and last phases (*p* = .001).

**Fig. 3:**
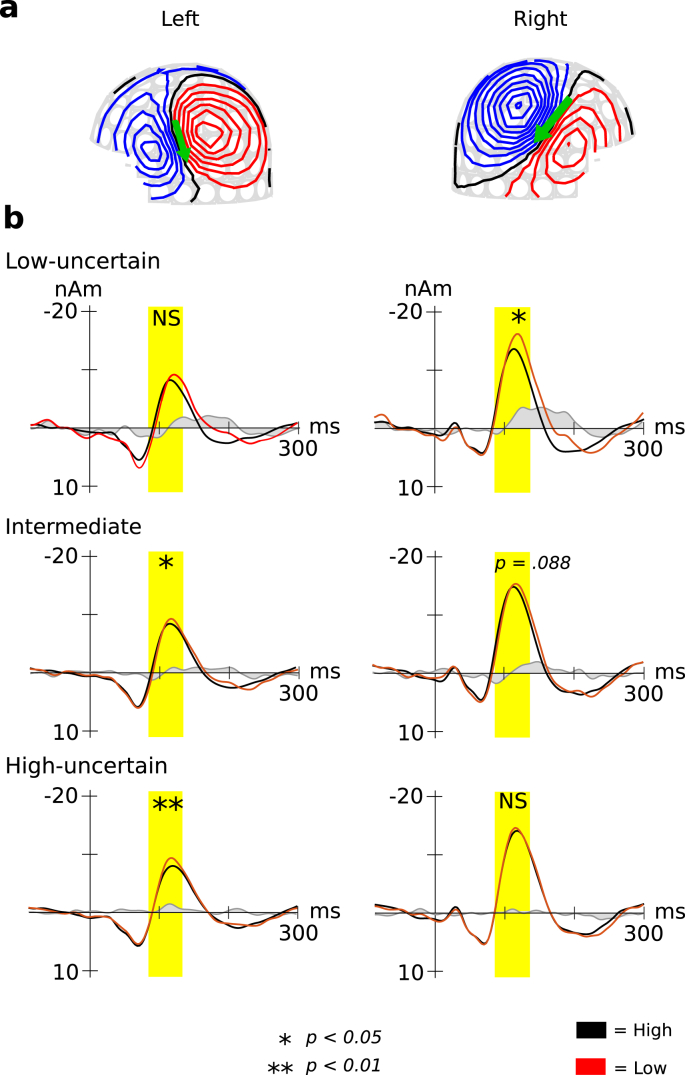
Grand-averaged source-strength waveforms for the N1m responses to tones (N = 13). **A** Isofield contour maps for N1m are shown for each hemisphere. Outflux (red lines) and influx (blue lines) are stepped by 20 fT. The green arrows represent ECDs. **B** The three tone sequences with different TP ratios of 90:10, 80:20, and 67:33 were prepared as the low-uncertain (conditional entropy = 0.47), intermediate (conditional entropy = 0.72), and the high-uncertainty sequences (conditional entropy = 0.92), respectively. The responses in the left and right hemispheres are located on the left and right sides, respectively. The blue and red lines indicate higher and lower TP, respectively. The grey represents difference between higher and lower TP. (For interpretation of the references to colour in this figure legend, the reader is referred to the Web version of this article.)

The hemisphere-probability-sequence interactions of the peak amplitudes were significant (*F*[2, 24] = 4.40, *p* = .024). In the low-uncertainty sequences in the right but not the left hemispheres, the peak amplitudes for the tones with low TP were significantly increased compared to those with high TP (*p* = .011) (Fig. 3). Conversely, in the highly uncertain sequences, in the left but not right hemispheres, the peak amplitudes for the tones with low TP were significantly increased compared to those with high TP (*p* = .005). In the intermediate sequences in the left hemisphere, the peak amplitudes for the tones with low TP were significantly increased compared to those with high TP (*p* = .012). Similar tendencies were also observed in the right hemisphere, although the difference was not significant (*p* = .088). In all three sequences, the peak amplitudes for each tone with high and low TP were significantly larger in the right hemisphere than in the left (low-uncertainty high: *p* = .001; low: *p* = .0001, intermediate high: *p* = .001; low: *p* = .002, high-uncertainty high: *p* = .0001; low: *p* = .002). The probability-sequence interactions of peak latencies were significant [*F* (2, 24) = 4.72, *p* = .019]. In the low-uncertainty, but not intermediate and highly uncertainty sequences, the peak latencies for the tones with low TP were significantly longer than those with high TP (*p* = .006; Fig. 1).

**Fig. 4:**
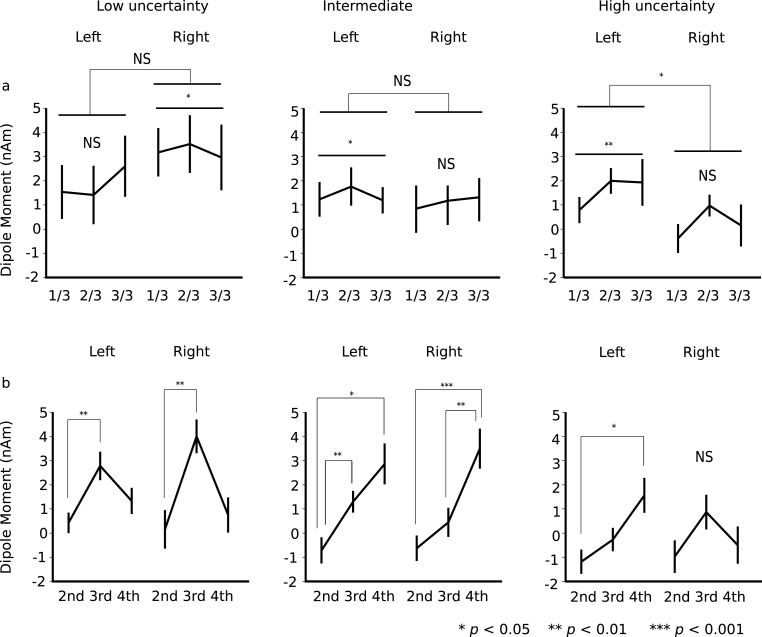
The differences in amplitudes. The peak amplitudes of a transition type (red in Fig. 2) were subtracted from those of the other transition type (blue in Fig. 2). The three tone sequences with different TP ratios of 90:10, 80:20, and 67:33 were prepared as the low-uncertain (conditional entropy = 0.47), intermediate (conditional entropy = 0.72), and the high-uncertain sequences (conditional entropy = 0.92), respectively.

Furthermore, we performed a 2 (hemisphere: right and left) × 3 (phase: first, middle, and last) × 3 (sequence: low-, intermediate-, and high-uncertainty sequences) repeated-measures ANOVA with differences in amplitudes that were subtracted from the peak N1m amplitudes for the tones with low from high TP. The hemisphere-sequence interactions were significant (*F*[2, 24] = 4.40, p = .024). In the high-uncertainty sequences, the differences in amplitudes were significantly larger in the left hemisphere than in the right hemisphere (*p* = .033; Fig. 4). No other significant differences were observed between groups.

### 3.2 The order of SL strategies

The three tone sequences were regarded not only as 1st- but also as 2nd-, 3rd-, and 4th-order Markov chains, and the responses to the tones with high (Fig. 2, blue) and low (Fig. 2, red) probabilities of occurrence in two transition patterns that differ only in the first tone were selectively averaged and compared, respectively. Then, we performed a 2 (hemisphere: right and left) × 3 (phase: first, middle, and last) × 3 (sequence: low-, intermediate-, and high-uncertainty sequences) repeated-measures ANOVA with differences in amplitudes that were subtracted from the peak N1m amplitudes for the tones with low (Fig. 2, red) from high (Fig. 2, blue) probabilities of occurrences in two transition patterns that were solely distinct first tones. The main order and sequence effects were significant (order *F*[2, 24] = 15.62, p = .0001; sequence *F*[2, 24] = 7.71, p = .003). The amplitude differences were significantly increased in the 3rd- and 4th-order Markov chains compared to the 2nd-order Markov chains (3rd: p = .001, 4th: p = .001). The differences in amplitudes were significantly increased in the low-uncertainty and intermediate sequences compared to the high-uncertainty sequences (low-uncertainty: p = .021, intermediate: p = .013). The sequence-order interactions were significant (*F*[4, 48] = 5.14, p = .002). In the low-uncertainty sequences, the difference amplitudes were significantly increased in the 3rd-order Markov chain, compared to in the 2nd- (p = .001) and 4th-order (p = .037) Markov chains. The findings may indicate that there is an optimal integration window for each uncertainty level. That is, a particular 'order’ that is being picked up depends on overall entropy (i.e., uncertainty). In the intermediate sequences, the differences in amplitudes were significantly increased in the 3rd- and 4th-order Markov chain compared to the 2nd-order Markov chains (3rd: p = .032, 4th: p = .001). The amplitude differences were significantly higher in the 4th-order Markov chain than in the 3rd-order Markov chains (p = .024). In the 3rd-order Markov chain, the differences in amplitudes were significantly increased in the low-uncertainty sequences compared to the intermediate (p = .049) and high-uncertainty (p = .015) sequences. In the 4th-order Markov chain, the differences in amplitudes were significantly increased in the intermediate-certainty sequence, compared to the low- (p = .027) and high-uncertainty (p = .004) sequences.

The hemisphere-sequence order interactions were significant (*F*[4, 48] = 4.16, p = .006; Fig. 4b). In the low-uncertainty sequences, the differences in amplitudes were significantly increased in the 3rd-order Markov chain, compared to the 2nd-order Markov chains in the left (p = .002) and right hemispheres (p = .001). In the intermediate sequences, the difference amplitudes in the left hemisphere were significantly increased in the 3rd- and 4th-order Markov chain, compared to the 2nd-order Markov chains (3rd: p = .008, 4th: p = .046). The amplitude differences in the right hemisphere were significantly higher in the 4th-order Markov chain than in the 2nd- (p = .001) and 3rd-order Markov chains (p = .001). In the high-uncertainty sequences, the differences in amplitudes in the left hemisphere were significantly increased in the 4th-order, compared to the 2nd-order Markov chains (p = .036). In the 3rd-order Markov chain, the differences in amplitudes in the left hemisphere were significantly increased in the low-uncertainty group compared to the high-uncertainty sequences (p = .017). In the 4th-order Markov chain, the differences in amplitudes in the right hemisphere were significantly increased in the intermediate, compared to the low-uncertainty (p = .005) and high-uncertainty sequences (p = .001). No other significant differences were observed between groups.

Further, we performed a 2 (hemisphere: right and left) × 3 (phase: first, middle, and last) × 2 (probability: high and low) × 3 (sequence: low-, intermediate-, and the high-uncertain sequences) × 3 (order: 2nd-, 3rd-, 4th-order Markov chains) repeated-measures ANOVA with the peak amplitude and latency of the source strength of N1m. All the results of the amplitudes and latencies are shown in the supplementary material.

## 4 Discussion

The present study examined how the uncertainty of a sequence modulates the neural effects of SL reflected in N1 components and whether the difference of uncertainty alters the order of SL strategies, using auditory sequences in which the uncertainty of sequential information is manipulated based on the conditional entropy. The results showed that stimuli with lower TPs elicited a stronger neural response than those with higher TPs (Fig. 3), as demonstrated by a number of previous studies based on predictive coding (e.g., Furl et al., 2011; Sanders et al., 2002; Tsogli et al., 2019). Furthermore, we found that participants adopted higher-order SL strategies in the high uncertainty sequence (Fig. 4b). These findings suggest the flexible alteration of “order” in SL strategies and that uncertainty may be an important factor that determines the order of SL strategies.

### 4.1 Interaction between prediction based on probability and uncertainty based on conditional entropy

Neural and behavioural studies have demonstrated that the degree of uncertainty fine-tunes the predictability of individual events in sequences during SL (Agres et al., 2018; Nastase et al., 2014), and modulates the neural mechanisms underlying SL (Harrison et al., 2006; Hasson, 2017; Okano et al., 2021). Hansen et al. (Hansen & Pearce, 2014) reported that the precision of uncertainty of melody is stronger among musicians than among non-musicians. This posits that long-term musical training produces a precise predictive model of music in the brain. The authors also suggested that high-uncertain information could impair the musician's advantageous predictive function. This indicates that prediction of each event based on TP and uncertainty of TP distribution interact with each other.

Indeed, the precision of uncertainty is an important role in hierarchical predictive processing of our brain (Friston, 2010). It has been generally thought that the prediction in our brain is controlled by the precision in higher hierarchy of neural processing. However, how this higher hierarchical neural processing of precision modulates prediction strategies remains unanswered. Importantly, from the perspective of information theory, the conditional entropy (i.e., uncertainty) is decreased by increasing the order of TP (Table 1). That is, increasing the order of TP has an advantage of reducing uncertainty of sequential information. The present study demonstrates the neural evidence of such an information-theoretical phenomenon: the brain predicts an event using higher-order SL strategies in a high uncertainty situation, and lower-order SL strategies in a low uncertain situation. Thus, the neural effects of SL (difference amplitudes between low and high TPs) in the low-uncertainty sequences were the larger in the 3rd-order strategies with conditional entropy of 0.45 than 4th-order strategies with conditional entropy of 0.44, while those in the intermediate sequence and high-uncertain sequences in the left hemisphere were the larger in the 4th-order than in the 3rd strategies (Fig. 4b). These findings may indicate that there is an optimal order for each uncertainty level and that the human brain has an ability to flexibly alter the order based on the uncertainty.

### 4.2 *”Order”* represents different levels of statistical learning

In the last decades, a body of researchers has examined how the human brain performs SL from the viewpoint of language acquisition, music perception, action, and so on. Most of these studies have focused on the specific order of SL strategies. One reason is that most studies have used the word segmentation paradigm for SL experiments (for review, see Daikoku, 2018c). This paradigm consists of a concatenation of pseudo-words, and the TPs of word-segmentation sequences are based on first-order statistical models. The word segmentation has generally been considered a mechanism for language acquisition in the early stages of language learning, even in infancy (J. R. Saffran et al., 1996). However, recent studies suggest that there are distinct stages of SL strategies in the context of memory system (Thiessen et al., 2013), chunking (Daikoku et al., 2017), and creativity (Wiggins, 2019). In fact, such a first-order SL is too simple to explain learning mechanisms of more complex sequential structures like real-world languages and music, and a higher-order SL is necessary for learning high uncertainty sequences. It has been suggested that a sequence with higher-TP produces stronger perceptual chunking as well as expectation or prediction. For example, a past study found that people rate higher-TP series as containing less unique elements (Tremblay et al.). A past neural study suggested that the brain performs word segmentation and word ordering (syntactic learning) using different "order" of SL strategies through information chunking (Daikoku et al., 2017). This suggests that such a flexible alteration of “order” is essential for explaining more complex SL functions.

**Fig. 5:**
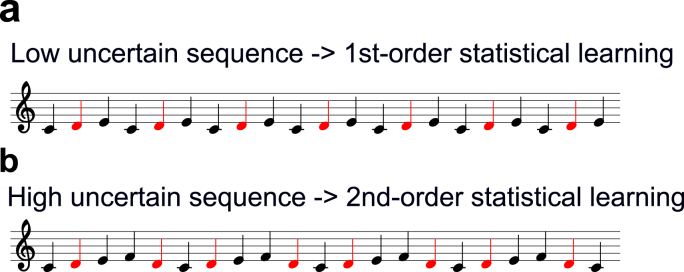
An example of different order of SL strategies. When persons conduct 1st-order SL of the above melody, a note after D is always E: 100% TP. Therefore, the 1st order is sufficient to learn this low uncertain sequence. In contrast, in the case of the below melody, it is difficult to predict a note after D only by the 1st-orde SL because D transitions C or E at the same TP (i.e., 50:50). However, if persons adopt 2nd-order SL strategy, we can easily predict an upcoming note. That is, a note after two notes C, D is always E while a note after two notes F, D is always C. In this way, 2nd-order SL strategy is more appropriate for the precise prediction of the below melody with high uncertainty while 1st-order SL strategy is enough for the prediction of the above melody with low uncertainty.

Fig. 5 shows an example about this processing. As shown, the above melody (Fig. 5a) is simpler than the below melody (Fig. 5b). The conditional entropy also shows that the above melody is of low uncertainty compared with the below melody. When persons conduct 1st-order SL of the above melody, a note after D is always E: 100% TP. Therefore, the 1st order is sufficient to learn this low uncertain sequence. In contrast, in the case of the below melody, it is difficult to predict a note after D only by the 1st-orde SL because D transitions C or E at the same TP (i.e., 50:50). However, if persons adopt 2nd-order SL strategy, we can easily predict an upcoming note. That is, a note after two notes C, D is always E while a note after two notes F, D is always C. In this way, 2nd-order SL strategy is more appropriate for the precise prediction of the below melody with high uncertainty while 1st-order SL strategy is enough for the prediction of the above melody with low uncertainty.

Evidences have suggested that individuals with developmental learning disorders, such as dyslexia (Du and Kelly, 2013) and amusia (Omigie et al., 2012, 2013; Omigie and Stewart, 2011) are impaired only for higher- but not lower-order SL. Computational modelling studies also indicate that individual differences in statistical knowledge gradually emerge from the lower-to the higher-order SL models (Daikoku, 2018b), and that statistical knowledge may shift from the lower-to the higher-order (deeper) hierarchy through experience (Daikoku, 2019). Thus, the order of SL strategies may shed a new light on understanding individual differences of SL ability.

### 4.3 Potential neural substrate of higher-order SL

The effects of low-uncertainty and high-uncertainty SL were pronounced in the right and left hemispheres, respectively. We also found that the amplitudes were larger in the right hemisphere than in the left hemisphere regardless of TP, which may be consistent with a large amount of evidence indicating that the right auditory cortex is specialised for spectral processing, such as pitches, whereas the left auditory cortex is specialised for temporal processing (Binder et al., 1997; Halpern and Zatorre, 1999; Zatorre et al., 1994). A previous study (Furl et al., 2011) suggested that the neural basis of the SL of auditory sequences with TP ratios of 10:90, which is the same as the low-uncertainty sequences in the present study, is associated with source activity in the right Brodmann area 39, posterior to the planum temporale, near the temporoparietal junction, and the superior temporal sulcus including parts of the middle and superior temporal gyri and the inferior angular gyrus. In contrast, another study reported that the SL of auditory sequences with TP ratios of approximately 64:14, which is close to the intermediate- and highly-uncertain sequences in the present study, is processed via pathways that connect superior temporal sources with the left inferior frontal gyrus in Brodmann's area 44 (Paraskevopoulos et al., 2017). This pathway includes Broca's area and has been considered to support the processing of hierarchical structure of auditory sequences in both language and music (Bahlmann et al., 2008; Maess et al., 2001; Molnar-Szakacs et al., 2005). Thus, these findings and our results suggest that the left hemisphere plays an important role in the neural basis of the processing of auditory sequences with higher uncertainty. In contrast, in the low-uncertainty, but not intermediate- and high-uncertainty sequences, the temporal profiles of the difference waveforms for stimuli with high and low TPs were similar to the MMN, especially from the viewpoint of the peak latencies (Fig. 3). The difference of waveforms in the low-uncertain sequences might be interpreted as an abstract MMN in the higher-order structure of the tone sequences (Herholz et al., 2009). That is, the neural responses in low-uncertainty sequences might partially share the neural substrates of MMN (Koelsch et al., 2016; Tsogli et al., 2019).

### 4.4 A novel approach to understand the order of SL strategies and the limitation of the present study

The results suggested that learning effects were detected in all of the sequences (Fig. 3) when comparing high and low TP within a transition ratio (first-order models in Fig. 2). However, when comparing high and low joint probabilities, regardless of the TP ratio (i.e., the number of co-occurrences shown in Fig. 2: blue and red), no SL effects were detected in the highly uncertain sequences (see Supplementary Material, Fig. 1g). However, it should be noted that the number of events (Fig. 2) in a model is the sum of the numbers of two events in the higher-order model. In other words, in the low- and intermediate-uncertainty sequences, only the high-TP event (first-order models in Fig. 2) was targeted when analysing 2nd-to 4th-order models (Fig. 4). Because the neural responses to tones were gradually attenuated with repetition due to the adaptation of auditory cortical neurons (supplementary material, Fig. 1c), it might be difficult to find SL effects only from the attenuated neural responses to stimuli with high TPs.

In this study, the sequence was presented with a SOA of 0.6 s, and the baseline for magnetic signals in each MEG channel was defined by the mean amplitude in the pre-stimulus period from −100 to 0 ms. This period has been used in most ERP/ERF studies on brain prediction including MMN (see Daikoku, 2018, Daikoku, 2018b for review). However, it is possible that such a long SOA may produce explicit predictions about a future stimulus. For example, a past study has revealed that long stimuli produce predictions in high TP series, and that these predictions can impact subsequent responses to stimuli with high vs. low-TP transitions (Cashdollar et al., 2017). Nevertheless, a short SOA is also problematic because ERP/ERF could be contaminated with neural activities after stimulus (such as offset response and later ERP/ERF). For example, numerous evidence showed SL is reflected with a later ERP/ERF component such as N400 (Cunillera et al., 2009; 2006; De Diego Balaguer et al., 2007; Sanders et al., 2002; Francois and Schön, 2011; 2013, 2014). Further, to detect large enough “N1” responses, which is the target in this study, epoch length around 600 ms is necessary (Koelsch et al., 2016; Tsogli et al., 2019). Thus, using the same baseline has the advantage to directly compare and discuss with previous findings of EEG/MEG on SL.

Another limitation is that in the low-uncertainty and intermediate sequences, the relationships between high and low joint probabilities are retained in each nth-order model (Fig. 2a and b, blue and red). In the highly uncertain sequences, however, the joint probabilities in the 3rd- and 4th-order models (Fig. 2c, blue and red) are the same between the compared transition patterns. Nevertheless, the SL effects in the highly uncertain sequences gradually increased from 2nd-to 4th-order models. One possible reason is that, as described above, in the low-uncertain and intermediate sequences, only the high-TP event (first-order models in Fig. 2) was targeted, whereas in the high-uncertainty sequences, both high- and low-TP events were targeted when analysing 2nd-to 4th-order models. Therefore, the neural responses analysed in high-uncertainty sequences may involve surprising effects based on prediction errors due to stimuli with low TPs. Nevertheless, to the best of our knowledge, the present study is the first to report neural correlates of higher-order as well as first-order SL and demonstrate that SL effects on neural responses to tones with a high TP in an order could be further distinguished by re-calculating as higher-order models.

## 5 Conclusions

We examined how the uncertainty of sequences modulates the neural effects of SL and whether the difference of uncertainty alters the order of SL strategies. The results found that participants adopted higher-order SL strategies in the high uncertainty sequence while they adopted a lower-order SL strategy in the low uncertainty sequence. These findings suggest that the human brain has an ability to flexibly alter the order based on the uncertainty. Thus, uncertainty may be an important factor that determines the “*order*” of SL strategies. The present study may shed new light on understanding individual differences of SL performance across different uncertain situation.

## CRediT authorship contribution statement

**Tatsuya Daikoku:** The experimental paradigms used in the present study were, created the paradigms, and TO recruited participants and collected the, Data curation, Formal analysis, both MEG and behavioural data, prepared figures, Writing – original draft. **Masato Yumoto:** The experimental paradigms used in the present study were, proposed, Methodology, for MEG, Formal analysis.

## Declaration of competing interest

The authors declare that they have no known competing financial interests or personal relationships that could have appeared to influence the work reported in this paper.

## Data Availability

Data will be made available on request.
