# Supplementary material for: Order of statistical learning depends on perceptive uncertainty
Source: Curr Res Neurobiol. 2023 Mar 1;4:100080. doi: 10.1016/j.crneur.2023.100080 (PMC10011828; doi:10.1016/j.crneur.2023.100080)
Supplement: Multimedia component 1 [file mmc1.docx]

**ANOVA results**

First, we performed a 2 (hemisphere: right and left) × 3 (phase: first, middle, and last) × 2 (probability: high and low) × 3 (sequence: low-uncertain, intermediate, and the high-uncertain Sequences) × 3 (order: 2nd-, 3rd-, 4th-order Markov chains) repeated-measures ANOVA with peak amplitude and latency of source-strength of N1m.

As a result, the main probability effect on the peak amplitudes and latencies were significant (amplitudes *F*[1, 12] = 8.37, p = .014; latencies *F*[1, 12] = 7.95, p = .015). The peak amplitudes and latencies for the tones with low TP were significantly larger and longer than those with high TP (Figure 1a and 6a). The main order effect on the peak amplitudes and latencies were significant (amplitudes *F*[2, 24] = 28.72, p = .0001; latencies *F*[2, 24] = 5.74, p = .009). The peak amplitudes were significantly increased in the 3rd- and 4th-order compared to 2nd-order Markov chains (3rd: p = .00001; 4th: p = .0001) and in the 4th-order than 3rd-order Markov chains (p = .040; Figure 1b). The peak latencies were significantly longer in the 4th-order than 2nd-order Markov chains (p = .048; Figure 2b). The main phase effect on the peak amplitudes and latencies were significant (amplitudes *F*[2, 24] = 26.29, *p* = .0001; latencies *F*[2, 24] = 18.63, *p* = .000). The peak amplitudes and latencies were significantly less and shorter in the middle and last than first phases (amplitude middle: *p* = 0.001; last: *p* = 0.0001, latency middle: *p* = 0.002; last: *p* = 0.002) (Figure 1c and 6c). The main hemisphere effect on the peak amplitudes and latencies were significant (amplitudes *F*[1, 12] = 24.885, p = .0001; latencies *F*[1, 12] = 5.11, p = .043). The peak amplitudes and latencies were significantly larger and shorter in right than left hemispheres (Figure 1d and 6d). The main sequence effect on the peak latencies was significant (*F*[2, 24] = 4.35, p = .024). However, no significance was detected in the post-hoc test.

The order-sequence interactions of the peak amplitudes were significant (*F*[4, 48] = 10.49, p = .0001). In the low-uncertain Sequences, the peak amplitudes were significantly increased in the 3rd-order, compared to the 2nd- and 4th-order Markov chains (2nd: p = .001; 4th: p = .048) (Figure 1e). In the intermediate and high-uncertain Sequences, the peak amplitudes were significantly increased in the 4th-order, compared to the 2nd- and 3rd-order Markov chains (intermediate 2nd: p = .0001; 3rd: p = .002, high-uncertain 2nd: p = .0001; 3rd: p = .030). The peak amplitudes were significantly increased in the 3rd-order, compared to the 2nd-order Markov chains (intermediate: p = .007; high-uncertain: p = .004). In the 4th-order Markov chain, the peak amplitudes were significantly increased in the high-uncertain compared to low-uncertain Sequences (p = .013, Figure 1e). The probability-order interactions of the peak amplitudes and latencies were significant (amplitudes *F*[2, 24] = 15.62, p = .0001; latencies *F*[2, 24] = 5.57, p = .010). The peak amplitudes for the tones with low TP were significantly increased in the 3rd- and 4th-order, compared to the 2nd-order Markov chains (3rd: p = .0001; 4th: p = .0001) (Figure 1f). The peak latencies for the tones with low TP were significantly increased in the 4th-order, compared to the 2nd- and 3rd-order Markov chains (2nd: p = .010; 3rd: p = .034) (Figure 2e). In the 3rd- and 4th-order Markov chains, the peak amplitudes for the tones with low TP were significantly increased compared to those with high TP (3rd: p = .001; 4th: p = .004). In the 4th-order Markov chains, the peak latencies for the tones with low TP were significantly longer than those with high TP (p = .002).

The probability-sequence interactions of the peak amplitudes were significant (*F*[2, 24] = 7.71, p = .003). In the low-uncertain and intermediate Sequences, the peak amplitudes for the tones with low TP were significantly increased compared to those with high TP (low-uncertain: p = .001; intermediate: p = .008). However, no significance was detected in the high-uncertain Sequences (Figure 1g). The phase-order interactions of the peak latencies were significant (*F*[4, 48] = 2.75, p = .039). In the last phase, the peak latencies were significantly longer in the 4th- than 2nd-order Markov chains (p = .041; Figure 2f). In each order of Markov chains, the peak latencies were significantly longer in the first than middle and last phases (2nd: middle: p = .003; last: p = .008, 3rd: middle: p = .001; last: p = .004, 4th: middle: p = .007; last: p = .001).

The probability-sequence-order interactions of the peak amplitudes were significant (*F*[4, 48] = 5.14, p = .002; Figure 1h). In the low-uncertain Sequences, the peak amplitudes for the tones with low TP were significantly larger in the 3rd-order Markov chain than in the 2nd- and 4th-order Markov chains (2nd: p = .001; 4th: p = .041). The peak amplitudes for the tones with high TP were significantly larger in the 2nd-order Markov chain than in the 3rd-order Markov chains (p = .012). In the intermediate Sequences, the peak amplitudes for the tones with low TP were significantly larger in the 3rd- and 4th-order Markov chain than in the 2nd-order Markov chains (3rd: p = .012; 4th: p = .0001), and larger in the 4th- than 3rd-order Markov chains (p = .006). In the high-uncertain Sequences, the peak amplitudes for the tones with low TP were significantly larger in the 3rd- and 4th-order Markov chain than in the 2nd-order Markov chains (3rd: p = .010; 4th: p = .002). The peak amplitudes for the tones with low TP were significantly increased compared to those with high TP in the 3rd-order Markov chain of the low-uncertain Markov chain (p = .0001) and the 4th-order Markov chain of the low-uncertain (p = .004) and intermediate Markov chains (p = .0001). In the 4th-order Markov chain, the peak amplitudes for the tones with high TP were significantly larger in the high-uncertain than in the low-uncertain Sequences (high: p = .048). The peak amplitudes for the tones with low TP were significantly larger in the intermediate and high-uncertain than in the low-uncertain Sequences (intermediate: p = .036; high-uncertain: p = .028).

The hemisphere-probability-sequence-order interactions of the peak amplitudes were significant (*F*[4, 48] = 4.16, p = .006; Figure 1i). In the low-uncertain Sequences, the peak amplitudes for the tones with low TP were significantly larger in the 3rd- than 2nd-order Markov chains in each hemisphere (left: p = .002; right: p = .002). The peak amplitudes for the tones with high TP were significantly larger in the 2nd- than 3rd-order Markov chains in each hemisphere (left: p = .042; right: p = .025) and in the 2nd- than 4th-order Markov chains in left hemisphere (p = .026). In the intermediate Sequences, the peak amplitudes for the tones with low TP were significantly larger in the 3rd- and 4th- than 2nd-order Markov chains in the left hemisphere (3rd: p = .007; 4th: p = .021), and larger in the 4th- than 2nd- and 3rd-order Markov chains in the right hemisphere (2nd: p = .0001; 3rd: p = .0001). In the high-uncertain Sequences, in the left hemisphere, the peak amplitudes for the tones with low TP were significantly larger in the 3rd- and 4th- than 2nd-order Markov chains (3rd: p = .005; 4th: p = .0001) and larger in the 4th- than 3rd-order Markov chains (p = .043). In the right hemisphere, the peak amplitudes for the tones with high TP were significantly larger in the 4th- than 2nd-order Markov chains (p = .012).

In the low-uncertain Sequences, the peak amplitudes for the tones with low TP were significantly increased compared to those with high TP in the 3rd-order Markov chains in the left and right hemispheres (left: p = .001; right: p = .002) and the 4th-order Markov chains in the left hemisphere (p = .006). In the intermediate Sequences, the peak amplitudes for the tones with low TP were significantly increased compared to those with high TP in the 3rd-order Markov chains in the left hemisphere (p = .009) and the 4th-order Markov chains in the left and right hemispheres (left: p = .015; right: p = .0001). In the high-uncertain Sequences, the peak amplitudes for the tones with high TP were significantly increased compared to those with low TP in the 2nd-order Markov chains in the left hemisphere (p = .018). In the 4th-order Markov chain, the peak amplitudes for the tones with low TP were significantly larger in the intermediate than low-uncertain Sequences (p = .011). The peak amplitudes for the tones with high TP were significantly larger in the high-uncertain than low-uncertain Sequences (p = .015). The peak amplitudes were significantly larger in the right than left hemispheres in the low-uncertain (high: 2nd: p=.001; 3rd: p=.001; 4th: p=.0001, low: 2nd: p=.005; 3rd: p=.001; 4th: p=.007), intermediate (high: 2nd: p=.0001; 3rd: p=.0001; 4th: p=.0001, low: 2nd: p=.001; 3rd: p=.002; 4th: p=.001), and high-uncertain Sequences (high: 2nd: p=.0001; 3rd: p=.0001; 4th: p=.0001, low: 2nd: p=.0001; 3rd: p=.0001; 4th: p=.004).

**
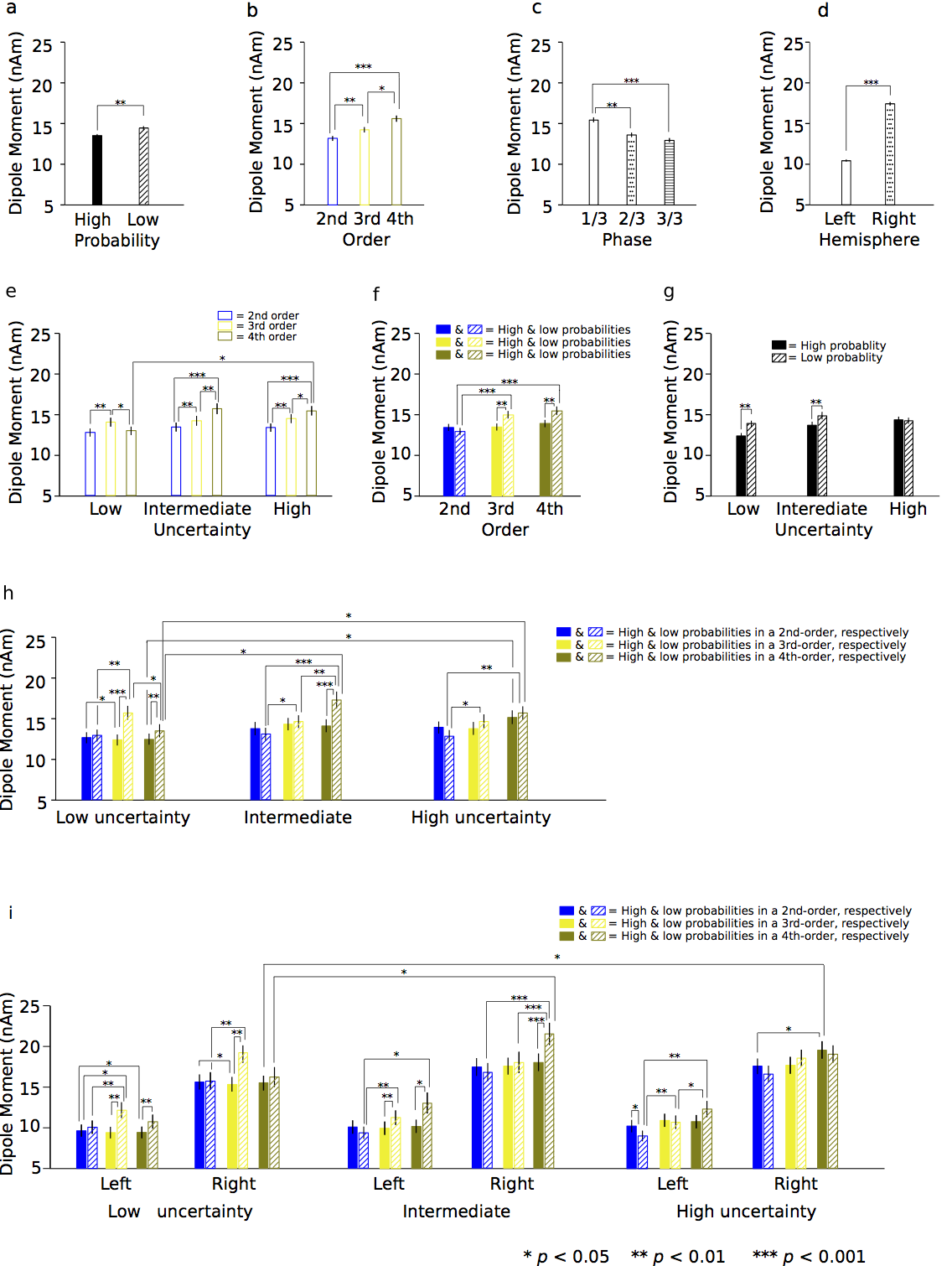
**

**Figure 1.** All of the significances of the peak amplitudes in N1m (N = 13). The repeated-measures ANOVA was conducted based on a 2 (hemisphere: right and left) × 3 (phase: 1/3, 2/3, and 3/3) × 2 (two types of transitions in Figure 2) × 3 (sequence: low-uncertain, intermediate, and the high-uncertain Sequences). The error bars indicate the standard error of the mean. The three tone sequences with different TP ratios of 90:10%, 80:20%, and 67:33% were prepared as the low-uncertain (conditional entropy = 0.47), intermediate (conditional entropy = 0.72), and the high-uncertain sequences (conditional entropy = 0.92), respectively.

**
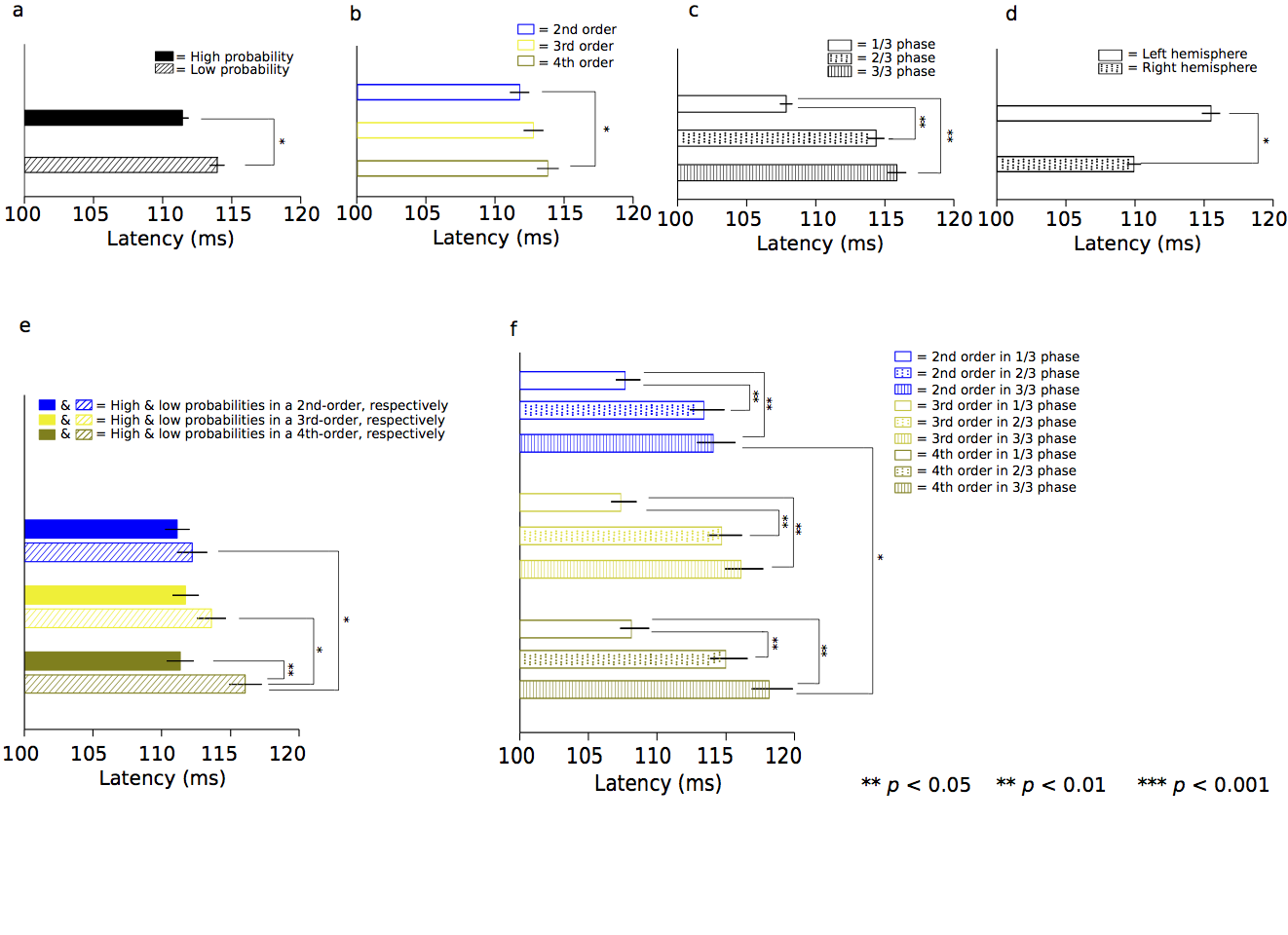
**

**Figure 2.** All of the significances of the peak latencies in N1m (N = 13). The repeated-measures ANOVA was conducted based on a 2 (hemisphere: right and left) × 3 (phase: 1/3, 2/3, and 3/3) × 2 (two types of transitions in Figure 2) × 3 (sequence: low-uncertain, intermediate, and the high-uncertain Sequences). The error bars indicate the standard error of the mean. The three tone sequences with different TP ratios of 90:10%, 80:20%, and 67:33% were prepared as the low-uncertain (conditional entropy = 0.47), intermediate (conditional entropy = 0.72), and the high-uncertain sequences (conditional entropy = 0.92), respectively.
