# Supplementary material for: Order of statistical learning depends on perceptive uncertainty
Source: Curr Res Neurobiol. 2023 Mar 1;4:100080. doi: 10.1016/j.crneur.2023.100080 (PMC10011828; doi:10.1016/j.crneur.2023.100080)
Supplement: Multimedia component 2 [file mmc2.pdf]

Peer Review Overview

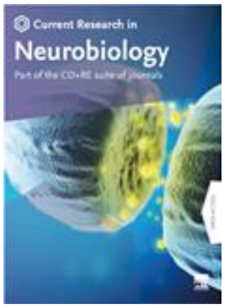

Manuscript Title: Perceptive Uncertainty Modulates the Order of Statistical Learning

Received	Apr 29, 2022
1st Decision	Sep 08, 2022
1st Revision Submitted	Oct 15, 2022
2nd Decision	Jan 09, 2023
2nd Revision Submitted	Feb 02, 2023
Accepted	Feb 06, 2023

1st Decision letter

Reference: CRNEUR-D-22-00044
Title: Perceptive Uncertainty Modulates the Order of Statistical Learning
Journal: Current Research in Neurobiology

Dear Professor Daikoku,

Thank you for submitting your manuscript to Current Research in Neurobiology.

I have completed my evaluation of your manuscript. The reviewers recommend reconsideration of your manuscript following major revision. I invite you to resubmit your manuscript after addressing the comments below. Please resubmit your revised manuscript by Nov 07, 2022.

When revising your manuscript, please consider all issues mentioned in the reviewers' comments carefully: please outline every change made in response to their comments and provide suitable rebuttals for any comments not addressed. Please note that your revised submission may need to be re-reviewed.

Current Research in Neurobiology values your contribution and I look forward to receiving your revised manuscript.

CRNEUR aims to be a unique, community-led journal, as highlighted in the Editorial Introduction. As part of this vision, we will be regularly seeking input from the scientific community and encourage you and your co-authors to take the survey.

Kind regards,

Kerry Walker, DPhil
Associate Editor
Current Research in Neurobiology

Comments from Editors and Reviewers:

Both reviewers have highlighted the merits of the submitted work, and agree that it provides new insights to the field of statistical learning. However, both reviewers also point out aspects of the manuscript that should be improved, added or changed prior to acceptance for publication. We invite the authors to make edits to their manuscript based on these reviews, and resubmit their edited manuscript with a point-by-point response to each reviewer comment, detailing how each suggestion has been addressed. Please ensure that the revised version of the manuscript includes line numbers throughout, as this is helpful for communication in the review stages.

In addition to addressing the reviewer comments below, I would ask the authors to reconsider Figure 2. It is excessively large (spanning 3 pages), and it is a collection of tables that are laborious to read. What is it that you want the reader to understand from looking at these matrices? Is there a more concise way to visually convey that information? Surely the reader is not expected to read and remember all these numbers. If a more easily understandable format is not possible, I'd suggest moving Figure 2 to the supplementary materials.

We look forward to receiving your revised manuscript.

Reviewer #1:

The authors report how statistical learning is reflected in MEG neural components using auditory sequences of tones. The main finding is that stimuli with lower transitional probabilities (i.e., with higher uncertainty) elicit stronger neural responses than those with higher TPs. Furthermore, when the uncertainty of the sequential structure is high (i.e., when the sequence has higher conditional entropy measured as the average uncertainty fo the outcome), this affects the statistical learning strategies, which the authors operationalize with the concept of "order". The results are interpreted in relation to how computational algorithms that model the order of statistical learning can contribute to our standing of SL strategies.

The paper makes a contribution to the literature on SL and particularly on our understanding of uncertainty and SL strategies. My major comments relate to the terminology used and how the MEG findings relate to the concept of "order". Specifically, i think the authors need to revise the definition of the concepts of "order" and "uncertainty" to make them more accessible to the reader. In addition, I don't fully understand how the concept of "order" is helpful to understand the recorded MEG signals since the main MEG results are related to the modulation of TPs. I include below my comments, which I hope the authors find helpful as they revise the manuscript

1. Please, provide comprehensive definition of the concepts of "uncertainty" and "order" early in the introduction. The first definition of uncertainty is in relation to the concept of conditional entropy, which the authors indicate that it is calculated from from TP distribution and interpreted as the average surprise or uncertainty of outcomes. This seems circular, please, provide the exact equation if needed. Similarly, the concept of "order" is defined on page 1 in relation to previous studies such as Du & Kelly, 2013; Omigie et al., 2012 etc. The authors refer to the concepts of higher and lower-order SL, but the term "order" is not defined.

2. The authors use the term "complexity" across the paper, but it is not defined. If it is used

interchangeably with the term "uncertainty", the authors should indicate this early in the paper. My impression is that the authors do not need to use the term complexity at all and that using uncertainty should be enough. It is a bit confusing to use both terms.

3. How are the MEG results related to the concept of order? If there is no relationship, then the title of the paper needs to be changed. My understanding is that a key finding is that stimuli with lower transitional probabilities (i.e., with higher uncertainty) elicit stronger neural responses than those with higher TPs .

4. The discussion needs a revision since it often reads as an introduction. For example, subsections 4.2 and 4.3 start with a description of prior literature and the main finding of the paper is only mentioned in the last few lines of the section. Please, refer to the main finding early in the subsection and then refer to other studies only if they help to understand the results of the paper. The focus of the discussion should be in what we can learn from the new results and not a description of prior findings.

5. As noted earlier, also in the Discussion, please indicate how modulation of the MEG signal relates to the concept of "order". It is unclear whether the concept of "order" is relevant to understand the MEG findings.

6. The authors refer across the paper to "the order of the SL", isn't there a noun missing? It seems that "the order of SL strategies", for example, would be more appropriate.

Reviewer #2:

This is an interesting and theoretically well-motivated paper examining an important aspect related to how humans use statistical regularities in input streams. The authors produce input streams using different types of 1st order Markov processes. These are processes where the probability of each stimulus depends only on the identity of stimulus that preceded it. Series where the transition constraints are less strong are more random (higher entropy) and series where the transition constraints are stricter are more regular/predictable (lower entropy). While prior work has studied neural processing of these systems as function of overall series entropy, or as function of the surprisal of each event given the immediate stimulus, this manuscript sets off from the observation that more regular series also allow a potential for what I would call 'chunking' or 'deep-prediction' across multiple items. E.g, if $P(b|a) = 0.9$, and $P(c|b) = 0.9$, $P(c|a)$ would be 0.81. It could therefore be that upon seeing 'a', some people would already predict 'bc'. So the processing of 'c' would be impacted not just by the immediately preceding event, but also, psychologically dependent on what occurred before 'b'. The authors refer to it as the 'order' of processing licensed by the statistical structure.

While the study is well motivated, and the experiment well designed and analyzed, there are multiple issues that require attention, some technical and some conceptual. For ease, I refer to them in the order by which they were encountered in the manuscript. It would help if a revision was submitted with page and line numbers to allow better reference.

1. the mathematical and conceptual definition of 'order' of a statistical learning process should be made clear in the introduction. Right now, the reader has to develop a sense for what the authors intend by looking at the figures they provide, which communicate the concept by example. I would recommend also to present an example, for instance, if the current stimulus is presented in uppercase, then responses to the Expected transition in the streak 'expected->expected->Expected' should be different

than when it is located in the streak 'unexpected->expected->Expected'. This would be evidence that responses to the current stimulus (Capitalized) is determined by a relatively long temporal integration window.

2. In section 2.2 it is written, "the probability of a forthcoming tone was statistically defined by the most recent two tones" with reference to Figure 1. I think Figure 1 might be wrong, or else the figure caption (which refers to columns and rows) is wrong. In any case, if the forthcoming tone's probability is the function of the most two recent ones, how is this a first order Markov process?

3. In section 2.2 it is written "Three distinct types of Markov chains were used in each of the three sequences and the use of Markov chains was counterbalanced across participants." The authors should state whether they verified that the transition probabilities of the series produced by the Markov process exactly matched the target probabilities? was there some simulation/selection step to assure a precise match?

4. In section 2.4.1 it is written "The baseline for magnetic signals in each MEG channel was defined by the mean amplitude in the pre-stimulus period from -100 to 0 ms." Given that the stimulus were presented for 300ms with an inter-stimulus-interval of 300ms, there was ample time for producing explicit predictions about a future stimulus. This could have produced different pre-stimulus baselines for series with high predictability and series with low predictability. If the baseline are different, then any normalization against baseline across conditions is problematic. This problem influences all analyses where activation patterns are directly compared across series with low/high transition probabilities. How do the authors deal with this issue?

5. In section 2.4.2.1 there is a reference to Figure 1, and the ANOVA is reported to include one factor referred to as "(probability: high and low in Figure 1)". Apart from the above mentioned problems with Figure 1, the experimental variables should be well defined enough to name them and refer to them without reference to a figure.

6. Similarly, in section 2.4.2.2 one variable in the ANOVA is referred to as "(transition: red and blue in Figure 2)". In this case as well, the variable must be defined as a conceptual factor. One possibility is to refer to it as a temporal integration window, based on minimal pairs of stimuli where the response to current tone differs only as function of a tone that was presented N-1, N-2 or N-3 elements ago. In addition, I was not able to find results reported for this analysis, though I did find the results for the other analysis in the section.

7. Section 3.2 "had solely distinct first tones"; needs rephrasing

8. In Section 3.2 it is written "In the low-uncertainty sequences, the difference amplitudes were significantly increased in the 3rd-order Markov chain, compared to in the 2nd- ($p = .001$) and 4th-order ($p = .037$) Markov chains" . Could the authors address the issue of whether their findings indicate that there is an optimal integration window for each uncertainty level (a particular 'order' that is being picked up depending on overall entropy)?

9. In section 4.1 there is an assumption that the human brain is trying to compute uncertainty alone. The authors can address the issue of whether the brain is trying to model uncertainty (entropy) or complexity (description length)

10. I found section 4.3 unrelated to the current experiment. It can be removed without loss of focus. The current paper does not address modality.

11. Caption of Figure 4 "They were subtracted the peak amplitudes of". Needs rephrasing

12. It is important that effort is made in the discussion to link the effects to some psychological mechanism: For example, is it related to predictions produced by chunking, backward-looking integration, or any other psychological process that could produce these effects.

13. I couldn't find files on the OSF site. I found the project, but not the files linked to it. The downloaded zip file was empty

1st Author Response Letter

Response to comments from Editors and Reviewers:

Comments from Reviewer 1:

We are extremely grateful for the Reviewer's insightful comments on our manuscript. We have studied the Reviewer's comments carefully and have made the necessary corrections in our paper. We believe that the comments have helped us significantly improve and refine the manuscript. Our responses to the Reviewer's comments are as follows.

Comment 1: Please, provide comprehensive definition of the concepts of "uncertainty" and "order" early in the introduction. The first definition of uncertainty is in relation to the concept of conditional entropy, which the authors indicate that it is calculated from from TP distribution and interpreted as the average surprise or uncertainty of outcomes. This seems circular, please, provide the exact equation if needed. Similarly, the concept of "order" is defined on page 1 in relation to previous studies such as Du & Kelly, 2013; Omigie et al., 2012 etc. The authors refer to the concepts of higher and lower-order SL, but the term "order" is not defined.

Response: We thank the reviewer for the thoughtful comments. As indicated, we revised the Introduction section and included the equation as follows. Further, we asked again to a professional English language editor for whole manuscript about English grammar and wording as attached below.

"This predictive processing underlies an innate function of the brain called statistical learning (SL) (Saffran et al., 1996). By implementing SL, the brain automatically encodes the n-th order transition probability (TP) of sequential information (Cleeremans et al., 1998; Perruchet & Pacton, 2006; Saffran et al., 1996; Daikoku, 2019) and grasps the uncertainty of TP distribution (Hasson, 2017). By SL of the sequential information, the brain predicts a subsequent event (en+1) based on the preceding events that have the length of "n" (en), written by a formula of P(en+1|en)."

"In particular, within the framework of SL, conditional entropy (H(Xn+1|Xn)) can be used and is calculated from the n-th order TP distribution. From a psychological viewpoint, a sequence with higher conditional entropy (i.e., a high uncertainty sequence) is regarded as sequential information that makes the human brain more difficult to implement SL and precisely predict individual events in sequences.

Evidence from previous studies has also revealed that the degree of uncertainty fine-tunes the predictability of individual events in sequences during SL (Agres et al., 2018; Nastase et al., 2014). Further, the neural mechanisms underlying SL can also be modulated by the degree of uncertainty (Harrison et. al, 2006; Hasson, 2017; Okano et al., 2021)."

"Both neural and modelling studies have suggested the importance of the "order" of SL strategies (Du & Kelly, 2013; Daikoku, 2018c, 2019). The concept of order corresponds to the length of the preceding events (en) to predict a subsequent event (en+1) as expressed in the formula of TP, P(en+1|en). The value of order (i.e., "n") represents the SL strategies of how long past events are referenced to predict the next. For example, if the current stimulus is presented in uppercase, then responses to the expected transition in the streak 'expected->expected- >Expected' should be different than when it is located in the streak 'unexpected->expected->Expected'. More simply, if an individual learned these sequences by SL based on first-order TP (hereafter, first-order SL), they would not be able to distinguish between the two because the preceding one event and predicted event are the same as each other (expected->Expected). However, if a person learned by second-order SL, the first event in the preceding two events is "'expected" for the former ('expected->expected->Expected') and "unexpected" for the latter ('unexpected->expected->Expected'), so the person can distinguish between them. This would be evidence that responses to the current stimulus (Capitalised) is determined by a relatively long temporal integration window or higher-order SL."

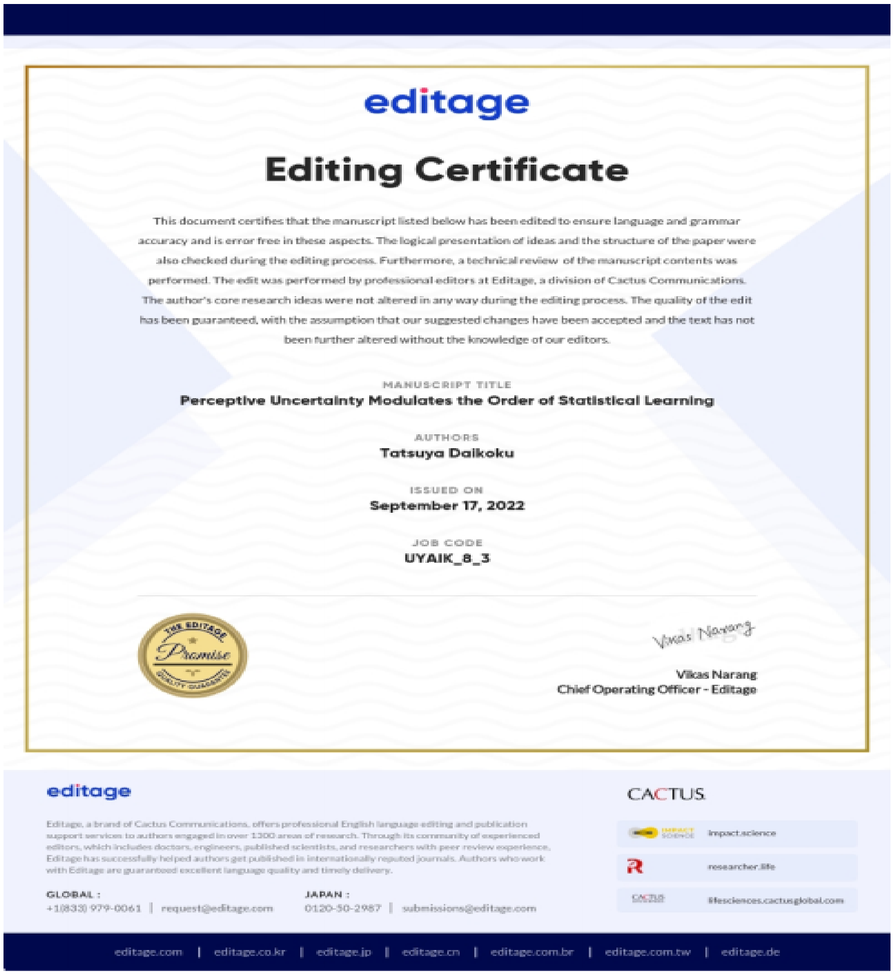
editage

Editing Certificate

This document certifies that the manuscript listed below has been edited to ensure language and grammar accuracy and is error free in these aspects. The logical presentation of ideas and the structure of the paper were also checked during the editing process. Furthermore, a technical review of the manuscript contents was performed. The edit was performed by professional editors at Editage, a division of Cactus Communications. The author's core research ideas were not altered in any way during the editing process. The quality of the edit has been guaranteed, with the assumption that our suggested changes have been accepted and the text has not been further altered without the knowledge of our editors.

MANUSCRIPT TITLE
Perceptive Uncertainty Modulates the Order of Statistical Learning

AUTHORS
Tatsuya Daikoku

ISSUED ON
September 17, 2022

JOB CODE
UYAIK_8_3

THE EDITAGE Promise QUALITY GUARANTEE

Vikas Narang
Vikas Narang
Chief Operating Officer - Editage

editage

Editage, a brand of Cactus Communications, offers professional English language editing and publication support services to authors engaged in over 1300 areas of research. Through its community of experienced editors, which includes doctors, engineers, published scientists, and researchers with peer review experience, Editage has successfully helped authors get published in internationally reputed journals. Authors who work with Editage are guaranteed excellent language quality and timely delivery.

GLOBAL :
+1(833) 979-0061 | request@editage.com

JAPAN :
0120-50-2987 | submissions@editage.com

CACTUS

impact.science

researcher.life

lifesciences.cactusglobal.com

editage.com | editage.co.kr | editage.jp | editage.cn | editage.com.br | editage.com.tw | editage.de

Comment 2: The authors use the term "complexity" across the paper, but it is not defined. If it is used interchangeably with the term "uncertainty", the authors should indicate this early in the paper. My impression is that the authors do not need to use the term complexity at all and that using uncertainty should be enough. It is a bit confusing to use both terms.

Response: Thank you very much for the pertinent comments. "Complexity" was used interchangeably with the term "uncertainty". As the reviewer indicated, using complexity is a bit confusing to potential readers. Therefore, we have only used "uncertainty" throughout the manuscript. These corrections are indicated in red text in the manuscript.

Comment 3: How are the MEG results related to the concept of order? If there is no relationship, then the title of the paper needs to be changed. My understanding is that a key finding is that stimuli with lower transitional probabilities (i.e., with higher uncertainty) elicit stronger neural responses than those with higher TPs.

Response: We thank the reviewer for the thoughtful comments. As the reviewer stated, the most important finding of this study is that stimuli with lower transitional probabilities (i.e., with higher uncertainty) elicit stronger neural responses than those with higher TPs. Similarly, our MEG results also suggested the importance of "order": when the uncertainty of the sequential structure was higher, the order of the SL strategies model increased. We discussed about these implications more deeply in the Discussion section as follows.

"Furthermore, we found that participants adopted higher-order SL strategies in the high uncertainty sequence (Figure 4b). These findings suggest the flexible alteration of "order" in SL strategies and that uncertainty may be an important factor that determines the order of SL strategies."

"The present study demonstrates the neural evidence of such an information-theoretical phenomenon: the brain predicts an event using higher-order SL strategies in a high uncertainty situation, and lower-order SL strategies in a low uncertain situation. Thus, the neural effects of SL (difference amplitudes between low and high TPs) in the low-uncertainty sequences were the larger in the 3rd-order strategies with conditional entropy of 0.45 than 4th-order strategies with conditional entropy of 0.44, while those in the intermediate sequence and highuncertain sequences in the left hemisphere were the larger in the 4th-order than in the 3rd strategies (Figure 4b). These findings may indicate that there is an optimal order for each uncertainty level and that the human brain has an ability to flexibly alter the order based on the uncertainty."

Comment 4: The discussion needs a revision since it often reads as an introduction. For example, subsections 4.2 and 4.3 start with a description of prior literature and the main finding of the paper is only mentioned in the last few lines of the section. Please, refer to the main finding early in the subsection and then refer to other studies only if they help to understand the results of the paper. The focus of the discussion should be in what we can learn from the new results and not a description of prior findings.

Response: We thank the reviewer for the pertinent comment. As indicated, we have drastically revised the Discussion section as follows. Further, we have revised section 4.2 and excluded the section 4.3 because we thought that this topic (modality) was not directly related with our study.

"4. Discussion

The present study examined how the uncertainty of a sequence modulates the neural effects of SL reflected in N1 components and whether the difference of uncertainty alters the order of SL strategies, using auditory sequences in which the uncertainty of sequential information is manipulated based on the conditional entropy. The results showed that stimuli with lower TPs elicited a stronger neural response than those with higher TPs (Figure 3), as demonstrated by a number of previous studies based on predictive coding (e.g., Furl et al., 2011; Sanders et al., 2002; Tsogli et al., 2019). Furthermore, we found that participants adopted higher-order SL strategies in the high uncertainty sequence (Figure 4b). These findings suggest the flexible alteration of "order" in SL strategies and that uncertainty may be an important factor that determines the order of SL strategies.

4.1. Interaction between prediction based on probability and uncertainty based on conditional entropy

Neural and behavioural studies have demonstrated that the degree of uncertainty fine-tunes the predictability of individual events in sequences during SL (Agres et al., 2018; Nastase et al., 2014), and modulates the neural mechanisms underlying SL (Harrison et. al, 2006; Hasson, 2017; Okano et al., 2021). Hansen et al. (Hansen & Pearce, 2014) reported that the precision of uncertainty of melody is stronger among musicians than among non-musicians. This posits that long-term musical training produces a precise predictive model of music in the brain. The authors also suggested that high-uncertain information could impair the musician's advantageous predictive function. This indicates that prediction of each event based on TP and uncertainty of TP distribution interact with each other.

Indeed, the precision of uncertainty is an important role in hierarchical predictive processing of our brain (Friston, 2010). It has been generally thought that the prediction in our brain is controlled by the precision in higher hierarchy of neural processing. However, how this higher hierarchical neural processing of precision modulates prediction strategies remains unanswered. Importantly, from the perspective of information theory, the conditional entropy (i.e., uncertainty) is decreased by increasing the order of TP (Table 1). That is, increasing the order of TP has an advantage of reducing uncertainty of sequential information. The present study demonstrates the neural evidence of such an information-theoretical phenomenon: the brain predicts an event using higher-order SL strategies in a high uncertainty situation, and lower-order SL strategies in a low uncertain situation. Thus, the neural effects of SL (difference amplitudes between low and high TPs) in the lowuncertainty sequences were the larger in the 3rd-order strategies with conditional entropy of 0.45 than 4th-order strategies with conditional entropy of 0.44, while those in the intermediate sequence and high-uncertain sequences in the left hemisphere were the larger in the 4th-order than in the 3rd strategies (Figure 4b). These findings may indicate that there is an optimal order for each uncertainty level and that the human brain has an ability to flexibly alter the order based on the uncertainty.

4.2. "Order" represents different levels of statistical learning

In the last decades, a body of researchers has examined how the human brain performs SL from the viewpoint of language acquisition, music perception, action, and so on. Most of these studies have focused on the specific order of SL strategies. One reason is that most studies have used the word segmentation paradigm for SL experiments (for review, see Daikoku, 2018c). This paradigm consists of a concatenation of pseudo-words, and the TPs of word-segmentation sequences are based on first-order

statistical models. The word segmentation has generally been considered a mechanism for language acquisition in the early stages of language learning, even in infancy (J. R. Saffran et al., 1996). However, recent studies suggest that there are distinct stages of SL strategies in the context of memory system (Thiessen et al., 2013), chunking (Daikoku, 2017), and creativity (Wiggins, 2018). In fact, such a first-order SL is too simple to explain learning mechanisms of more complex sequential structures like real-world languages and music, and a higher-order SL is necessary for learning high uncertainty sequences. A past neural study suggested that the brain performs word segmentation and word ordering (syntactic learning) using different "order" of SL strategies through information chunking (Daikoku et al., 2017). This suggests that such a flexible alteration of "order" is essential for explaining more complex SL functions.

Figure 5 shows an example about this processing. As shown, the above melody (Figure 5a) is simpler than the below melody (Figure 5b). The conditional entropy also shows that the above melody is of low uncertainty compared with the below melody. When persons conduct 1st-order SL of the above melody, a note after D is always E: 100% TP. Therefore, the 1st order is sufficient to learn this low uncertain sequence. In contrast, in the case of the below melody, it is difficult to predict a note after D only by the 1st-orde SL because D transitions C or E at the same TP (i.e., 50:50). However, if persons adopt 2nd-order SL strategy, we can easily predict an upcoming note. That is, a note after two notes C, D is always E while a note after two notes F, D is always C. In this way, 2nd-order SL strategy is more appropriate for the precise prediction of the below melody with high uncertainty while 1st-order SL strategy is enough for the prediction of the above melody with low uncertainty. Evidences have suggested that individuals with developmental learning disorders, such as dyslexia (Du & Kelly, 2013) and amusia (Omigie et al., 2012; Omigie et al., 2013; Omigie & Stewart, 2011) are impaired only for higher- but not lower-order SL. Computational modelling studies also indicate that individual differences in statistical knowledge gradually emerge from the lower- to the higher-order SL models (Daikoku, 2018b), and that statistical knowledge may shift from the lower- to the higher-order (deeper) hierarchy through experience (Daikoku, 2019). Thus, the order of SL strategies may shed a new light on understanding individual differences of SL ability.

4.3. Potential neural substrate of higher-order SL

The effects of low-uncertainty and high-uncertainty SL were pronounced in the right and left hemispheres, respectively. We also found that the amplitudes were larger in the right hemisphere than in the left hemisphere regardless of TP, which may be consistent with a large amount of evidence indicating that the right auditory cortex is specialised for spectral processing, such as pitches, whereas the left auditory cortex is specialised for temporal processing (Binder et al., 1997; Halpern & Zatorre, 1999; Zatorre et al., 1994). A previous study (Furl et al., 2011) suggested that the neural basis of the SL of auditory sequences with TP ratios of 10:90, which is the same as the low-uncertainty sequences in the present study, is associated with source activity in the right Brodmann area 39, posterior to the planum temporale, near the temporoparietal junction, and the superior temporal sulcus including parts of the middle and superior temporal gyri and the inferior angular gyrus. In contrast, another study reported that the SL of auditory sequences with TP ratios of approximately 64:14, which is close to the intermediate- and highly-uncertain sequences in the present study, is processed via pathways that connect superior temporal sources with the left inferior frontal gyrus in Brodmann's area 44 (Paraskevopoulos et al, 2017). This pathway includes Broca's area and has been considered to support the processing of hierarchical structure of auditory sequences in both language and music (Bahlmann et

al., 2008; Maess et al., 2001; Molnar-Szakacs et al., 2004). Thus, these findings and our results suggest that the left hemisphere plays an important role in the neural basis of the processing of auditory sequences with higher uncertainty. In contrast, in the low-uncertainty, but not intermediate- and high-uncertainty sequences, the temporal profiles of the difference waveforms for stimuli with high and low TPs were similar to the MMN, especially from the viewpoint of the peak latencies (Figure 3). The difference of waveforms in the low-uncertain sequences might be interpreted as an abstract MMN in the higher-order structure of the tone sequences (Herholz et al., 2009). That is, the neural responses in low-uncertainty sequences might partially share the neural substrates of MMN (Koelsch et al., 2016; Tsogli et al., 2019).

4.4. A novel approach to understand the order of SL strategies and the limitation of the present study

The results suggested that learning effects were detected in all of the sequences (Figure 3) when comparing high and low TP within a transition ratio (first-order models in Figure 2). However, when comparing high and low joint probabilities, regardless of the TP ratio (i.e., the number of co-occurrences shown in Figure 2: blue and red), no SL effects were detected in the highly uncertain sequences (see Supplementary Material, Figure 1g). However, it should be noted that the number of events (Figure 2) in a model is the sum of the numbers of two events in the higher-order model. In other words, in the low- and intermediate-uncertainty sequences, only the high-TP event (first-order models in Figure 2) was targeted when analysing 2nd- to 4th-order models (Figure 4). Because the neural responses to tones were gradually attenuated with repetition due to the adaptation of auditory cortical neurons (supplementary material, Figure 1c), it might be difficult to find SL effects only from the attenuated neural responses to stimuli with high TPs.

In this study, the sequence was presented with a SOA of 0.6 second, and the baseline for magnetic signals in each MEG channel was defined by the mean amplitude in the pre-stimulus period from -100 to 0 ms. This period has been used in most ERP/ERF studies on brain prediction including MMN (see Daikoku, 2018 for review). However, it is possible that such a long SOA may produce explicit predictions about a future stimulus. Nevertheless, a short SOA is also problematic because ERP/ERF could be contaminated with neural activities after stimulus (such as offset response and later ERP/ERF). For example, numerous evidence showed SL is reflected with a later ERP/ERF component such as N400 (Cunillera et al., 2009, 2006; De Diego Balaguer et al., 2007; Sanders et al., 2002; Francois et al., 2011; 2013; 2014). Further, to detect large enough “N1” responses, which is the target in this study, epoch length around 600 ms is necessary (Koelsch et al., 2016; Tsogli et al., 2019). Thus, using the same baseline has the advantage to directly compare and discuss with previous findings of EEG/MEG on SL.

Another limitation is that in the low-uncertainty and intermediate sequences, the relationships between high and low joint probabilities are retained in each nth-order model (Figure 2a and 2b, blue and red). In the highly uncertain sequences, however, the joint probabilities in the 3rd- and 4th-order models (Figure 2c, blue and red) are the same between the compared transition patterns. Nevertheless, the SL effects in the highly uncertain sequences gradually increased from 2nd- to 4th-order models. One possible reason is that, as described above, in the low-uncertain and intermediate sequences, only the high-TP event (first-order models in figure 2) was targeted, whereas in the high-uncertainty sequences, both high- and low-TP events were targeted when analysing 2nd- to 4th-order models. Therefore, the neural responses analysed in high-uncertainty sequences may involve surprising effects based on prediction errors due to stimuli with low TPs. Nevertheless, to the best of our knowledge, the present study is the

first to report neural correlates of higher-order as well as first-order SL and demonstrate that SL effects on neural responses to tones with a high TP in an order could be further distinguished by re-calculating as higher-order models."

Comment 5: As noted earlier, also in the Discussion, please indicate how modulation of the MEG signal relates to the concept of "order". It is unclear whether the concept of "order" is relevant to understand the MEG findings.

Response: The MEG results suggested the importance of "order": when the uncertainty of the sequential structure was higher, the order of the SL strategies model increased. We revised the Discussion section so that potential readers can clearly understand the concept of "order" as follows.

"The present study demonstrates the neural evidence of such an information-theoretical phenomenon: the brain predicts an event using higher-order SL strategies in a high uncertainty situation, and lower-order SL strategies in a low uncertain situation. Thus, the neural effects of SL (difference amplitudes between low and high TPs) in the low-uncertainty sequences were the larger in the 3rd-order strategies with conditional entropy of 0.45 than 4th-order strategies with conditional entropy of 0.44, while those in the intermediate sequence and highuncertain sequences in the left hemisphere were the larger in the 4th-order than in the 3rd strategies (Figure 4b). These findings may indicate that there is an optimal order for each uncertainty level and that the human brain has an ability to flexibly alter the order based on the uncertainty."

Comment 6: The authors refer across the paper to "the order of the SL", isn't there a noun missing? It seems that "the order of SL strategies", for example, would be more appropriate.

Response: Thank you very much for bringing to our notice this important point. As suggested, we have modified from "the order of the SL" to "the order of SL strategies" throughout the manuscript. These corrections are indicated in red text in the manuscript.

Comments from Reviewer 2

We wish to express our strong appreciation to the Reviewer for the insightful comments on our manuscript. We have studied the Reviewer's comments very carefully and have made necessary corrections. We feel the comments have helped us significantly improve the manuscript.

Comment 1: the mathematical and conceptual definition of 'order' of a statistical learning process should be made clear in the introduction. Right now, the reader has to develop a sense for what the authors intend by looking at the figures they provide, which communicate the concept by example. I would recommend also to present an example, for instance, if the current stimulus is presented in uppercase, then responses to the Expected transition in the streak 'expected->expected->Expected' should be different than when it is located in the streak 'unexpected->expected->Expected'. This would be evidence that responses to the current stimulus (Capitalized) is determined by a relatively long temporal integration window.

Response: We thank the reviewer for the thoughtful comments. As indicated, we clarified the definition of uncertainty in the Introduction section as follows. Further, we also explained about the terminology of order. To make the added information clearer, we also included these equation as follows. Finally, we

asked again to a professional English language editor for whole manuscript about English grammar and wording as attached below.

Introduction seciton

“This predictive processing underlies an innate function of the brain called statistical learning (SL) (Saffran et al., 1996). By implementing SL, the brain automatically encodes the n-th order transition probability (TP) of sequential information (Cleeremans et al., 1998; Perruchet & Pacton, 2006; Saffran et al., 1996; Daikoku, 2019) and grasps the uncertainty of TP distribution (Hasson, 2017). By SL of the sequential information, the brain predicts a subsequent event (en+1) based on the preceding events that have the length of “n” (en), written by a formula of P(en+1|en).”

“In particular, within the framework of SL, conditional entropy (H(Xn+1|Xn)) can be used and is calculated from the n-th order TP distribution. From a psychological viewpoint, a sequence with higher conditional entropy (i.e., a high uncertainty sequence) is regarded as sequential information that makes the human brain more difficult to implement SL and precisely predict individual events in sequences. Evidence from previous studies has also revealed that the degree of uncertainty fine-tunes the predictability of individual events in sequences during SL (Agres et al., 2018; Nastase et al., 2014). Further, the neural mechanisms underlying SL can also be modulated by the degree of uncertainty (Harrison et. al, 2006; Hasson, 2017; Okano et al., 2021).”

“Both neural and modelling studies have suggested the importance of the “order” of SL strategies (Du & Kelly, 2013; Daikoku, 2018c, 2019). The concept of order corresponds to the length of the preceding events (en) to predict a subsequent event (en+1) as expressed in the formula of TP, P(en+1|en). The value of order (i.e., "n") represents the SL strategies of how long past events are referenced to predict the next. For example, if the current stimulus is presented in uppercase, then responses to the expected transition in the streak 'expected->expected- >Expected' should be different than when it is located in the streak 'unexpected->expected->Expected'. More simply, if an individual learned these sequences by SL based on first-order TP (hereafter, first-order SL), they would not be able to distinguish between the two because the preceding one event and predicted event are the same as each other (expected->Expected). However, if a person learned by second-order SL, the first event in the preceding two events is "'expected" for the former ('expected->expected->Expected') and "unexpected" for the latter ('unexpected->expected->Expected'), so the person can distinguish between them. This would be evidence that responses to the current stimulus (Capitalised) is determined by a relatively long temporal integration window or higher-order SL.”

Discussion section

“Figure 5 shows an example about this processing. As shown, the above melody (Figure 5a) is simpler than the below melody (Figure 5b). The conditional entropy also shows that the above melody is of low uncertainty compared with the below melody. When persons conduct 1st-order SL of the above melody, a note after D is always E: 100% TP. Therefore, the 1st order is sufficient to learn this low uncertain sequence. In contrast, in the case of the below melody, it is difficult to predict a note after D only by the 1st-orde SL because D transitions C or E at the same TP (i.e., 50:50). However, if persons adopt 2nd-order SL strategy, we can easily predict an upcoming note. That is, a note after two notes C, D is always E while a note after two notes F, D is always C. In this way, 2nd-order SL strategy is more appropriate for

the precise prediction of the below melody with high uncertainty while 1st-order SL strategy is enough for the prediction of the above melody with low uncertainty.”

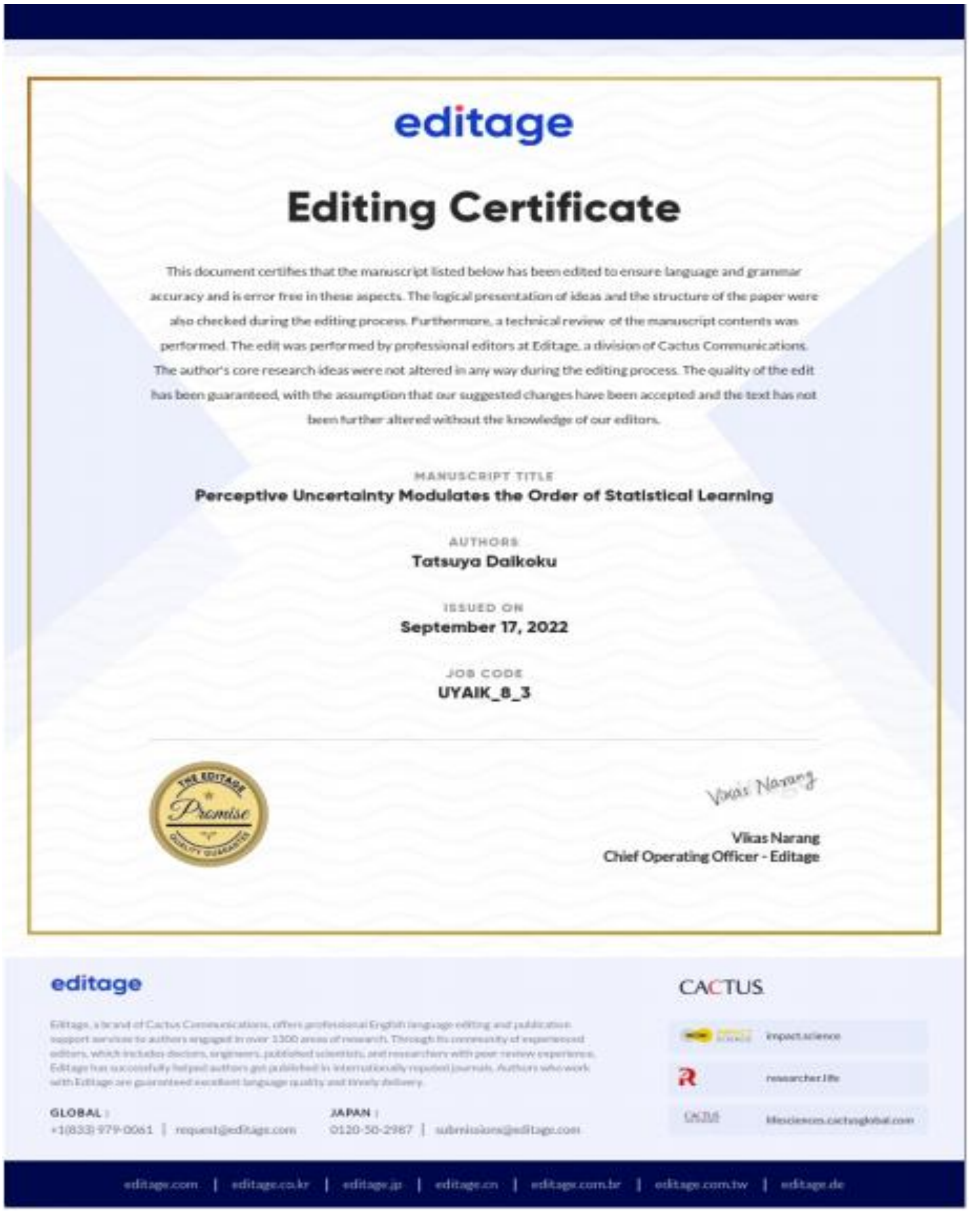

editage

Editing Certificate

This document certifies that the manuscript listed below has been edited to ensure language and grammar accuracy and is error free in these aspects. The logical presentation of ideas and the structure of the paper were also checked during the editing process. Furthermore, a technical review of the manuscript contents was performed. The edit was performed by professional editors at Editage, a division of Cactus Communications. The author's core research ideas were not altered in any way during the editing process. The quality of the edit has been guaranteed, with the assumption that our suggested changes have been accepted and the text has not been further altered without the knowledge of our editors.

MANUSCRIPT TITLE
Perceptive Uncertainty Modulates the Order of Statistical Learning

AUTHORS
Tatsuya Daikoku

ISSUED ON
September 17, 2022

JOB CODE
UYAIK_8_3

THE EDITAGE Promise QUALITY GUARANTEE

Vikas Narang
Vikas Narang
Chief Operating Officer - Editage

editage

CACTUS

impact.science
researcher.life

GLOBAL : +1(833) 979-0061 | request@editage.com
JAPAN : 0120-50-2987 | submissions@editage.com

editage.com | editage.co.kr | editage.jp | editage.cn | editage.com.br | editage.com.tw | editage.de

Comment 2:

In section 2.2 it is written, "the probability of a forthcoming tone was statistically defined by the most recent two tones" with reference to Figure 1. I think Figure 1 might be wrong, or else the figure caption (which refers to columns and rows) is wrong. In any case, if the forthcoming tone's probability is the function of the most two recent ones, how is this a first order Markov process?

Response: Thank you for bringing to our notice the incorrect sentence and caption. As indicated, we have revised the sentences of Section 2.2 and the caption of Figure 1 as follows.

Section 2.2

“The order of pure tones was defined according to first-order Markov models (i.e., bi-gram model) with the constraint that the probability of a forthcoming tone was statistically defined by the most recent tone (Figure 1). The two-tone sequences with different TP ratios of 90:10, 80:20, and 67:33, in which each conditional entropy in the probability distribution of sequences was 0.47, 0.72, and 0.92, respectively, were prepared as low-, intermediate-, and high-uncertainty sequences, respectively.”

Caption of figure 1

“Figure 1. The transition diagram of the Markov chain used in the study. The tone could follow the most recent tone based on a higher or lower TP ratio. The two-tone sequences with different TP ratios of 90:10, 80:20, and 67:33 were prepared as the low-uncertain (conditional entropy = 0.47), intermediate (conditional entropy = 0.72), and the high-uncertain sequences (conditional entropy = 0.92), respectively.”

Comment 3: In section 2.2 it is written "Three distinct types of Markov chains were used in each of the three sequences and the use of Markov chains was counterbalanced across participants." The authors should state whether they verified that the transition probabilities of the series produced by the Markov process exactly matched the target probabilities? was there some simulation/selection step to assure a precise match?

Response: We thank the reviewer for this important point. The sequences were generated to match the target probabilities, which we have described in the section 2.2. We have also uploaded the file that describes information of these sequences in OSF site and stated it as follows.

“The TPs of the sequences produced by these Markov processes exactly matched the target TPs and the conditional entropy (see OSF site: https://osf.io/xcr97/ for the information of three sequences).”

Comment 4: In section 2.4.1 it is written "The baseline for magnetic signals in each MEG channel was defined by the mean amplitude in the pre-stimulus period from -100 to 0 ms." Given that the stimulus were presented for 300ms with an inter-stimulus-interval of 300ms, there was ample time for producing explicit predictions about a future stimulus. This could have produced different pre-stimulus baselines for series with high predictability and series with low predictability. If the baseline are different, then any normalization against baseline across conditions is problematic. This problem influences all analyses where activation patterns are directly compared across series with low/high transition probabilities. How do the authors deal with this issue?

Response: We thank the reviewer for the thoughtful comments. Typically, most of ERP/ERF studies on brain prediction, including MMN, has used the same baseline between low and high probability (see, Daikoku, 2018 for review). Therefore, using the same baseline has the advantage to directly compare and discuss with the previous findings of EEG/MEG on statistical learning. Contrastingly, I partially agree with the opinion that SOA of 600ms may produce explicit predictions about a future stimulus. However, short SOA is also problematic because ERP/ERF could be contaminated with neural activities after stimulus (such as offset response and later ERP/ERF). For example, numerous evidence showed statistical learning is reflected with a later ERP/ERF component such as N400 (Cunillera et al., 2009, 2006; De Diego Balaguer et al., 2007; Sanders et al., 2002; Francois et al., 2011; 2013; 2014). This means, if we use a short SOA like 300ms, ERP/ERF could be contaminated with neural activity of one previous

stimuli. Further, to detect large enough “N1” responses, which is the target in this study, epoch length around 600 ms is necessary (Koelsch et al., 2016; Tsogli et al., 2019). We described the above information as a limitation in the Discussion section as follows.

“In this study, the sequence was presented with a SOA of 0.6 second, and the baseline for magnetic signals in each MEG channel was defined by the mean amplitude in the pre-stimulus period from -100 to 0 ms. This period has been used in most ERP/ERF studies on brain prediction including MMN (see Daikoku, 2018 for review). However, it is possible that such a long SOA may produce explicit predictions about a future stimulus. Nevertheless, a short SOA is also problematic because ERP/ERF could be contaminated with neural activities after stimulus (such as offset response and later ERP/ERF). For example, numerous evidence showed SL is reflected with a later ERP/ERF component such as N400 (Cunillera et al., 2009, 2006; De Diego Balaguer et al., 2007; Sanders et al., 2002; Francois et al., 2011; 2013; 2014). Further, to detect large enough “N1” responses, which is the target in this study, epoch length around 600 ms is necessary (Koelsch et al., 2016; Tsogli et al., 2019). Thus, using the same baseline has the advantage to directly compare and discuss with previous findings of EEG/MEG on SL.”

Comment 5: In section 2.4.2.1 there is a reference to Figure 1, and the ANOVA is reported to include one factor referred to as "(probability: high and low in Figure 1)". Apart from the above mentioned problems with Figure 1, the experimental variables should be well defined enough to name them and refer to them without reference to a figure.

Response: Thank you for the pertinent comment. As indicated, we have clearly defined the variables in section 2.4.2.1 as follows.

“We performed a 2 (“Hemisphere”: right and left) × 3 (“Phase”: first, middle, and last) × 2 (“Probability”: high and low) × 3 (“Sequence”: low-, intermediate-, and high-uncertainty sequences) repeated-measures analysis of variance (ANOVA) with the peak amplitude and latency of the source strength of N1m. Furthermore, we performed a 2 (“Hemisphere”: right and left) × 3 (“Phase”: first, middle, and last) × 3 (“Sequence”: low-, intermediate-, and high-uncertainty sequences) repeated-measures ANOVA with differences in amplitudes that were subtracted from the peak amplitudes for tones with high TP from those with low TP.”

Comment 6: Similarly, in section 2.4.2.2 one variable in the ANOVA is referred to as "(transition: red and blue in Figure 2)". In this case as well, the variable must be defined as a conceptual factor. One possibility is to refer to it as a temporal integration window, based on minimal pairs of stimuli where the response to current tone differs only as function of a tone that was presented N-1, N-2 or N-3 elements ago. In addition, I was not able to find results reported for this analysis, though I did find the results for the other analysis in the section.

Response: We thank you for the comment. I realized that this is “probability” because the probability of occurrence of the series is high (blue in Figure 2) or low (red in Figure 2), regardless of the order of Markov process. We have revised the variables in section 2.4.2.2 as follows. The results of this analysis were reported in the section of 3.2. The order of SL strategies.

“a 2 (“Hemisphere”: right and left) × 3 (“Phase”: first, middle, and last) × 2 (“Probability”: high and low) × 3 (sequence: low-, intermediate-, and high-uncertainty sequences) repeated-measures ANOVA”

"a 2 ("Hemisphere": right and left) × 3 ("Phase": first, middle, and last) × 3 ("Sequence": low-, intermediate-, and high-uncertainty sequences) × 3 ("Order": 2nd-, 3rd-, 4th-order Markov chains) repeated-measures ANOVA"

Comment 7: Section 3.2 "had solely distinct first tones"; needs rephrasing

Response: Thank you for the pertinent comment. As indicated, we have rephrased it in section 3.2 as follows. "The responses to the tones with high (Figure 2, blue) and low (Figure 2, red) probabilities of occurrence in two transition patterns that differ only in the first tone were selectively averaged and compared, respectively."

Comment 8: In Section 3.2 it is written "In the low-uncertainty sequences, the difference amplitudes were significantly increased in the 3rd-order Markov chain, compared to in the 2nd- ($p = .001$) and 4th-order ($p = .037$) Markov chains" . Could the authors address the issue of whether their findings indicate that there is an optimal integration window for each uncertainty level (a particular 'order' that is being picked up depending on overall entropy)?

Response: We thank the reviewer for the thoughtful comments. We have specified it in section 3.2. and the Discussion section as follows.

Section 3.2 "The findings may indicate that there is an optimal integration window for each uncertainty level. That is, a particular 'order' that is being picked up depends on overall entropy (i.e., uncertainty)."

Comment 9: In section 4.1 there is an assumption that the human brain is trying to compute uncertainty alone. The authors can address the issue of whether the brain is trying to model uncertainty (entropy) or complexity (description length)

Response: In this paper, "complexity" almost corresponds to uncertainty. Therefore, we have only used the term "uncertainty". These corrections are indicated in red text in the manuscript.

Comment 10: I found section 4.3 unrelated to the current experiment. It can be removed without loss of focus. The current paper does not address modality.

Response: We thank the reviewer for the thoughtful comments. I agree with the opinion that section 4.3. can be removed without loss of focus and is unrelated with the results of this paper. We have excluded it.

Comment 11: Caption of Figure 4 "They were subtracted the peak amplitudes of". Needs rephrasing

Response: Thank you for letting me know the wrong expression of English. We revised the caption of Figure 4 as follows. "The peak amplitudes of a transition type (red in Figure 2) were subtracted from those of the other transition type (blue in Figure 2)."

Comment 12: It is important that effort is made in the discussion to link the effects to some psychological mechanism: For example, is it related to predictions produced by chunking, backward-looking integration, or any other psychological process that could produce these effects.

Response: We thank the reviewer for the insightful suggestions.

Comment 13: I couldn't find files on the OSF site. I found the project, but not the files linked to it. The downloaded zip file was empty

Response: We were not aware of this issue and apologize for inconvenience this may have caused. We have uploaded all the data and sound stimuli in the OSF site (DOI 10.17605/OSF.IO/XCR97).

2nd Decision letter

Reference: CRNEUR-D-22-00044
Title: Perceptive Uncertainty Modulates the Order of Statistical Learning
Journal: Current Research in Neurobiology

Dear Professor Daikoku,

Thank you for submitting your manuscript to Current Research in Neurobiology.

Please accept our apologies for the delay in getting this decision to you. We were struggling to get a second reviewer report, but are now able to proceed.

I am delighted to be able to now suggest only minor revisions to your manuscript. One of the reviewers recommend reconsideration of your manuscript following minor revision, and their review is copied below. I invite you to resubmit your manuscript after addressing their comments, which the reviewer and I believe will be straightforward. Please resubmit your revised manuscript by Feb 08, 2023.

When revising your manuscript, please consider all issues mentioned in the reviewers' comments carefully: please outline every change made in response to their comments and provide suitable rebuttals for any comments not addressed. Please note that your revised submission may need to be re-reviewed.

Current Research in Neurobiology values your contribution and I look forward to receiving your revised manuscript.

Kind regards,

Kerry Walker, DPhil
Associate Editor
Current Research in Neurobiology

Comments from Editors and Reviewers:

Reviewer comments:

The authors addressed all the comments satisfactorily. The manuscript is very much improved.

I have the following comments that address minor points.

1. Regarding the inter-stimulus interval of 600ms, the authors write "However, it is possible that such a long SOA may produce explicit predictions about a future stimulus". There is actually evidence that long stimuli produce predictions in high TP series, and that these predictions can impact subsequent responses to high vs. low-TP transitions. See Cashdollar et al. https://doi.org/10.1093/cercor/bhw138

2. grammar : From a psychological viewpoint, a sequence with higher conditional entropy (i.e., a high uncertainty sequence) is regarded as sequential information that makes the human brain more difficult to implement SL.." rewrite "makes it more difficult for the human brain "

3. grammar : The value of order (i.e., "n") represents the SL strategies of how long past events are referenced to predict the next.
I'd rephrase 'long' --> 'many'

4. There's at least one other study that shows that high-TP series produce stronger perceptual chunking, which might be operating here as well as mediator of 'prediction'. Tremblay et al. show that higher-TP series produce a behavioral result where listeners rate series as containing less unique elements. http://dx.doi.org/10.1016/j.neuroimage.2012.10.055

2nd Author Response Letter

Response to comments from Editors and Reviewers:

Comments from Reviewer :

We are extremely grateful for the Reviewer's insightful comments on our manuscript. We have studied the Reviewer's comments carefully and have made the necessary corrections in our paper. We believe that the comments have helped us significantly improve and refine the manuscript. Our responses to the Reviewer's comments are as follows.

Comment 1:

Regarding the inter-stimulus interval of 600ms, the authors write "However, it is possible that such a long SOA may produce explicit predictions about a future stimulus". There is actually evidence that long stimuli produce predictions in high TP series, and that these predictions can impact subsequent responses to high vs. low-TP transitions. See Cashdollar et al. https://doi.org/10.1093/cercor/bhw138

Response:

Thank you very much for the pertinent comment and showing really important paper. We have cited this paper and discussed them in the Discussion Section as follows.

"For example, a past study has revealed that long stimuli produce predictions in high TP series, and that these predictions can impact subsequent responses to stimuli with high vs. low-TP transitions (Cashdollar et al., 2017)."

Comment 2:

grammar : From a psychological viewpoint, a sequence with higher conditional entropy (i.e., a high uncertainty sequence) is regarded as sequential information that makes the human brain more difficult to implement SL.." rewrite "makes it more difficult for the human brain "

Response:

We thank the reviewer for the comment. We've modified as follows.

"that makes it more difficult for the human brain"

Comment 3:

grammar : The value of order (i.e., "n") represents the SL strategies of how long past events are referenced to predict the next.

I'd rephrase 'long' --> 'many'

Response:

Thank you for the comment. As indicated, we've modified as follows.

"The value of order (i.e., "*n*") represents the SL strategies of how many past events are referenced to predict the next."

Comment 4:

There's at least one other study that shows that high-TP series produce stronger perceptual chunking, which might be operating here as well as mediator of 'prediction'. Tremblay et al. show that higher-TP series produce a behavioral result where listeners rate series as containing less unique elements. http://dx.doi.org/10.1016/j.neuroimage.2012.10.055

Response:

Thank you very much for the pertinent comment and letting us know the important study! We have cited it and discussed in the Discussion Section as follows.

"It has been suggested that a sequence with higher-TP produces stronger perceptual chunking as well as expectation or prediction. For example, a past study found that people rate higher-TP series as containing less unique elements (Tremblay et al)."

Accept Letter

Dear Professor Daikoku,

Thank you for submitting your manuscript to Current Research in Neurobiology.

I am pleased to inform you that your manuscript has been accepted for publication.

Your accepted manuscript will now be transferred to our production department. We will create a proof which you will be asked to check, and you will also be asked to complete a number of online forms required for publication. If we need additional information from you during the production process, we will contact you directly.

We appreciate and value your contribution to Current Research in Neurobiology. We regularly invite authors of recently published manuscript to participate in the peer review process. If you were not already part of the journal's reviewer pool, you have now been added to it. We look forward to your continued participation in our journal, and we hope you will consider us again for future submissions.

CRNEUR aims to be a unique, community-led journal, as highlighted in the Editorial Introduction. As part of this vision, we will be regularly seeking input from the scientific community and encourage you and your co-authors to take the survey.

We would also like to invite you to take part in our CRNEUR Author Question & Answer (Q&A), which could get published alongside your article and help to promote it. We suspect you might have an interesting story of perseverance or team work that was required for the research study to complete, or a diversity of perspectives that you might share, as a way of inspiring others about neuroscience.

Kind regards,

Kerry Walker, DPhil
Associate Editor
Current Research in Neurobiology

-------- *End of Review Comments* --------
